# Adjusting for time‐varying confounders in survival analysis using structural nested cumulative survival time models

**DOI:** 10.1111/biom.13158

**Published:** 2019-11-07

**Authors:** Shaun Seaman, Oliver Dukes, Ruth Keogh, Stijn Vansteelandt

**Affiliations:** ^1^ MRC Biostatistics Unit, University of Cambridge Institute of Public Health Cambridge UK; ^2^ Department of Applied Mathematics, Computer Science and Statistics Ghent University Ghent Belgium; ^3^ London School of Hygiene and Tropical Medicine London UK

**Keywords:** accelerated failure time model, Aalen's additive model, G‐estimation, marginal structural model, survival data

## Abstract

Accounting for time‐varying confounding when assessing the causal effects of time‐varying exposures on survival time is challenging. Standard survival methods that incorporate time‐varying confounders as covariates generally yield biased effect estimates. Estimators using weighting by inverse probability of exposure can be unstable when confounders are highly predictive of exposure or the exposure is continuous. Structural nested accelerated failure time models (AFTMs) require artificial recensoring, which can cause estimation difficulties. Here, we introduce the structural nested cumulative survival time model (SNCSTM). This model assumes that intervening to set exposure at time t to zero has an additive effect on the subsequent conditional hazard given exposure and confounder histories when all subsequent exposures have already been set to zero. We show how to fit it using standard software for generalized linear models and describe two more efficient, double robust, closed‐form estimators. All three estimators avoid the artificial recensoring of AFTMs and the instability of estimators that use weighting by the inverse probability of exposure. We examine the performance of our estimators using a simulation study and illustrate their use on data from the UK Cystic Fibrosis Registry. The SNCSTM is compared with a recently proposed structural nested cumulative failure time model, and several advantages of the former are identified.

## 1 INTRODUCTION

Observational studies that attempt to assess the effect of a time‐varying exposure on a survival outcome typically suffer from time‐varying confounding bias. Such bias is the result of time‐varying factors that both influence exposure and are associated with survival, thereby distorting the association between the two. For example, studies of the effect of hospital‐acquired pneumonia on time to death (since hospital admission) in critically ill patients are confounded by disease severity, because disease severity influences susceptibility to pneumonia infection and is strongly associated with mortality (Bekaert *et al.*, 2010). Time‐varying confounders (eg, disease severity) are often affected by earlier exposures (eg, pneumonia infection). This induces feedback relationships between exposures and confounders over time that cannot be untangled via traditional survival analysis regression methods that adjust for time‐varying covariates, such as a history of exposure and confounders, at each timepoint (Robins *et al.*, 2000). The reason for this is twofold. First, such adjustment procedures eliminate the indirect effects of early exposures on survival that are mediated through those confounders. For example, it would be undesirable to eliminate the effects of hospital‐acquired pneumonia on survival that are mediated through disease severity, as scientific interest is primarily in the overall effect of infection. Second, such adjustment procedures are prone to collider‐stratification biases that can render exposure and outcome dependent even in the absence of an exposure effect. See Daniel *et al.* (2013) for a review of these difficulties.

Time‐varying confounding has received much attention in the causal inference literature. For survival time outcomes, the two main approaches are based on structural nested accelerated failure time models (AFTMs) (Robins and Tsiatis, 1991; Robins and Greenland, 1994) and marginal structural models (MSMs) (Robins *et al.*, 2000). The latter approach is more popular, because of its greater simplicity and flexibility. In particular, accounting for noninformative censoring in MSMs does not, unlike in structural nested AFTMs, require an “artificial recensoring” procedure in which originally uncensored subjects may become censored. Avoiding this recensoring is advantageous, because recensoring causes information loss, which can result in poor estimators and difficulties solving the estimating equations (Joffe *et al.*, 2012). However, fitting MSMs relies on inverse weighting by the probability of exposure, which has it own drawback: estimators prone to large finite‐sample bias and variance when confounders are highly predictive of the exposure, or when the exposure is continuous or discrete with many levels.

More recently, Young *et al.* (2010) and Picciotto *et al.* (2012) proposed a new class of discrete‐time structural nested cumulative failure time models, which parameterize the effect of the exposure at each time t on the outcome at each later time in terms of the ratio of two (possibly) counterfactual cumulative failure risks at that later time under exposure regimes that differ only at time t. Their procedure has the desirable properties of structural nested AFTMs—viz. by avoiding inverse probability weighting, it handles continuous exposures without estimators being subject to large bias and variance, and it allows modeling of effect modification by time‐varying covariates—while avoiding the need for artificial recensoring.

Here, we use developments by Martinussen *et al.* (2011) and Dukes *et al.* (2019) (hereafter DMTV). The former showed how to adjust for time‐varying confounding when the effects of exposure and confounders are parameterized on the additive hazard scale. They focused on the simple setting where interest is in estimating the direct effect of a binary baseline exposure on a survival outcome, that is, the effect not mediated by a given intermediate variable, and where there are no baseline confounders. DMTV proposed an additive hazards model for the effect of a baseline exposure on survival time conditional on baseline confounders and derived the efficient score when (as assumed by Martinussen *et al*. 2011) the confounders act additively on the hazard; this additivity assumption is not needed for consistency of their estimators.

Here, we propose a novel class of semiparametric structural nested cumulative survival time models (SNCSTMs), of which the models of Martinussen *et al.* (2011) and DMTV are special cases, and propose three estimators of its parameters. Our model allows baseline and time‐varying confounders, binary or continuous exposure, any number of exposure measurement times and the option of constraining exposure effects to be common at different times; it does not parameterize the effects of confounders on the baseline hazard. It also allows investigation of exposure effect modification by time‐varying factors. The SNCSTM is closely related to Picciotto *et al.*’s model, and our estimators share the forementioned desirable properties of the latter. The SNCSTM generalizes Picciotto *et al*.’s model to continuous time and parameterizes relative survival risks instead of failure risks. Our approach has several advantages over that of Picciotto *et al*. One of our estimators (method 1) can be calculated using GLM software. Our other two estimators (methods 2 and 3) are more efficient, double robust and available in closed form. All three estimators automatically handle random censoring. Also, because of being parameterized in continuous time, SNCSTMs can handle irregular measurement times and allow the interpretation of parameters in terms of hazards.

We define notation and state fundamental assumptions in Section 2. A simple version of our SNCSTM is introduced in Section 3. In Section 4, we propose three methods for estimating its parameters. The general SNCSTM is described in Section 5. In Section 6, we discuss random censoring. A simulation study is described in Section 7. Section 8 describes an analysis of data from the UK Cystic Fibrosis (CF) Registry, looking at the effect of treatment with DNase on survival in people with CF. We conclude with a discussion in Section 9.

## 2 NOTATION AND ASSUMPTIONS

Consider a study in which, for each of n subjects, a time‐varying exposure and vector of possibly time‐varying confounders are measured at time zero and at up to K follow‐up visits. Until Section 5 we assume the follow‐up times are regular, that is, the same for all individuals, and (for notational simplicity) are 1,2,…,K, and that all individuals are administratively censored at time K+1. Until Section 6 we assume there is no censoring apart from this administrative censoring. If visits are regular but not at times 1,…,K, or if administrative censoring occurs at a time different from K+1 or not at all, this can easily be accommodated by rescaling the time variable within each interval between consecutive visits.

Let $T_{i}$ denote individual i’s failure time, and $A_{ki}$ and $L_{ki}$ denote, respectively, his exposure and vector of confounders measured at time k (k=0,…,K). Let $R_{i}\left(t\right)=I\left(T_{i}⩾t\right)$ be the at‐risk indicator. If individual i fails before his kth visit, $A_{ki}$ and $L_{ki}$ are defined as zero. Let ${\bar{A}}_{ki}={\left(A_{0i},\text{…},A_{ki}\right)}^{⊤},{\bar{L}}_{ki}={\left(L_{0i},\text{…},L_{ki}\right)}^{⊤}$, and $A_{-1,i}\equiv \emptyset$. The causal ordering of the variables is $\left\{L_{0},A_{0},T\wedge 1,L_{1},A_{1},T\wedge 2,\text{…},L_{K},A_{K},T\wedge \left(K+1\right)\right\}$, where x∧y means the minimum of x and y.

Define $T_{i}\left({\bar{A}}_{ki},0\right)$ as individual i’s (possibly) counterfactual failure time that would have applied if his exposures up to the kth visit had been as observed and his exposures from the (k+1)th visit onward had been set to zero by an intervention. We make the consistency assumption that $T_{i}=T_{i}\left({\bar{A}}_{ki},0\right)$ with probability one for individuals with $A_{k+1,i}=\text{⋯}=A_{Ki}=0$. Note $T_{i}\left({\bar{A}}_{k-1,i},0\right)⩾k$ if and only if $T_{i}\left({\bar{A}}_{li},0\right)⩾k$ for all l=k,…,K, that is, intervening on an exposure can only affect survival after the time of that exposure. It follows that events $\left\{T_{i}⩾t\right\}$ and $\left\{T_{i}\left(A_{ki},0\right)⩾t\right\}$ are equivalent when t∈[k,k+1). We assume $\left({\bar{A}}_{Ki},{\bar{L}}_{Ki},T_{i}\right)$ (i=1,…,n) are i.i.d. and henceforth omit the subscript i unless needed.

We make the following sequential no unmeasured confounders assumption (NUC): $T\left({\bar{A}}_{k-1},0\right)⊥\;⊥A_{k}|{\bar{L}}_{k},{Ā}_{k-1},T⩾k\left(k=0,\text{…},K\right)$ (Robins, 1986). That is, among individuals who are still alive (or event‐free) at time k, the assigned exposure $A_{k}$ at time k may depend on ${\bar{L}}_{k}$ and ${\bar{A}}_{k-1}$, but given these, has no residual dependence on the remaining lifetime that would apply if all future exposures were set to zero.

## 3 STRUCTURAL NESTED CUMULATIVE SURVIVAL TIME MODEL

We first introduce a simple version of the SNCSTM that does not allow for exposure effect modification. The more general SNCSTM is described in Section 5.

For each k=0,…,K, let $M_{k}$ be the model defined by the restriction

(1) $$\frac{P\left\{T\left({\bar{A}}_{k},0\right)⩾t|{\bar{A}}_{k},{\bar{L}}_{k},T⩾k\right\}}{P\left\{T\left({\bar{A}}_{k-1},0\right)⩾t|{\bar{A}}_{k},{\bar{L}}_{k},T⩾k\right\}}=\text{exp}\left\{-A_{k}v_{k}{\left(t\right)}^{⊤}\psi_{k}\right\},$$

∀t⩾k, where $\psi_{k}={\left(\psi_{k\left(k\right)},\psi_{k\left(k+1\right)},\text{…},\psi_{k\left(K\right)}\right)}^{⊤}$ is a vector of K−k+1 unknown parameters, and $v_{k}\left(t\right)$ equals ${\left(t-k,0,\text{…},0\right)}^{⊤}$ if t∈[k,k+1), equals ${\left(1,t-k-1,0,\text{…},0\right)}^{⊤}$ if t∈[k+1,k+2), and equals ${\left(1,1,t-k-2,0,\text{…},0\right)}^{⊤}$ if t∈[k+2,k+3), and so forth. So, for any $k⩽l⩽t<l+1,v_{k}{\left(t\right)}^{⊤}\psi_{k}$ equals $\psi_{k\left(k\right)}+\text{⋯}+\psi_{k\left(l-1\right)}+\psi_{k\left(l\right)}\left(t-l\right)$.

Equation (1) means that among the survivors in the population at the kth visit time, in the stratum defined by any given $\left({\bar{A}}_{k},{\bar{L}}_{k}\right)$ the proportion who survive to a later time t when exposures from visit k+1 onward (ie, $A_{k+1},\text{…},A_{K}$) have already been set to zero would be multiplied by $\text{exp}\left\{A_{k}v_{k}{\left(t\right)}^{⊤}\psi_{k}\right\}$ if exposure $A_{k}$ were also set to zero. Hence, $v_{k}{\left(t\right)}^{⊤}\psi_{k}$ is the (controlled) direct effect of $A_{k}$ on the probability of survival to time t given survival to visit k, that is, the effect of $A_{k}$ not mediated through the later exposures $A_{k+1},\text{…},A_{l}$. For example, if $\psi_{k\left(k\right)},\text{…},\psi_{k\left(K\right)}$ are all positive and $A_{k}>0$, then intervening to set $A_{k}=0$ is beneficial, that is, exposure is harmful. Conversely, if $\psi_{k\left(k\right)},\text{…},\psi_{k\left(K\right)}$ are all negative, exposure is beneficial. This SNCSTM assumes the direct effect $v_{k}{\left(t\right)}^{⊤}\psi_{k}$ is the same for any history $\left({\bar{A}}_{k-1},{\bar{L}}_{k}\right)$. In Section 5 we extend the SNCSTM to allow the effect to depend on the history.

By taking logs of each side of Equation (1) and differentiating with respect to t, it can be shown that Model $M_{k}$ can also be written as

(2) $$\;\begin{array}{ccc} & & E\left\{dN_{\left({\bar{A}}_{k-1},0\right)}\left(t\right)|{\bar{A}}_{k},{\bar{L}}_{k},T\left({\bar{A}}_{k-1},0\right)⩾t\right\}\\ & & =\;E\left\{dN_{\left({\bar{A}}_{k},0\right)}\left(t\right)|{\bar{A}}_{k},{\bar{L}}_{k},T\left({\bar{A}}_{k},0\right)⩾t\right\}-A_{k}\psi_{k\left(l\right)}dt \end{array}$$

for t∈[l,l+1) (with l=k,…,K), where $N_{\left({\bar{A}}_{k},0\right)}\left(t\right)=I\left\{T\left({\bar{A}}_{k},0\right)⩽t\right\}$ is the counting process for $T\left({\bar{A}}_{k},0\right)$. Equation (2) can be interpreted as follows. In a stratum defined by $\left({\bar{A}}_{k},{\bar{L}}_{k}\right)$ and T⩾k, the hazard of failure at any time between visits l and l+1 (l⩾k) when $A_{k+1},\text{…},A_{l}$ have already been set equal to zero would be reduced by $A_{k}\psi_{k\left(l\right)}$ if $A_{k}$ were also set to zero.

Note that Model $M_{k}$ treats $E\left\{dN_{\left({\bar{A}}_{k-1},0\right)}\left(t\right)|{\bar{A}}_{k},{\bar{L}}_{k},T\left({\bar{A}}_{k-1},0\right)⩾t\right\}$—which, by NUC, equals $E\left\{dN_{\left({\bar{A}}_{k-1},0\right)}\left(t\right)|{\bar{A}}_{k-1},{\bar{L}}_{k},T\left({\bar{A}}_{k-1},0\right)⩾t\right\}$—as a totally unspecified “baseline” hazard, rather than parameterizing its dependence on ${\bar{A}}_{k-1}$ and ${\bar{L}}_{k}$. One advantage of this is that the danger of incompatibility between Models $M_{0},\text{…},M_{K}$ is avoided. To illustrate this danger, suppose it were assumed that $E\left\{dN\left(t\right)|{\bar{A}}_{1},{\bar{L}}_{1},T⩾t\right\}=\phi_{10}\left(t\right)+\phi_{1A_{0}}\left(t\right)A_{0}+\phi_{1{\bar{L}}_{1}}{\left(t\right)}^{⊤}{\bar{L}}_{1}+\psi_{1\left(1\right)}A_{1}$ for all t⩾1. This, together with NUC, implies $M_{1}$ holds. However, it also implies a restriction on the association between $A_{0}$ and T, a restriction which might conflict with that of $M_{0}$. Such conflict would be the result of there being no coherent overall model.

## 4 ESTIMATION METHODS

In order to estimate $\psi_{k\left(l\right)}$, we introduce nuisance Models $A_{k}$ (k=0,…,K). Model $A_{k}$ is a generalized linear model (GLM) for $A_{k}$ given ${\bar{A}}_{k-1},{\bar{L}}_{k}$ and T⩾k with $g\left\{E\left(A_{k}|{\bar{A}}_{k-1},{\bar{L}}_{k},T⩾k\right)\right\}=\alpha_{k0}^{⊤}H_{k}$, where $\alpha_{k0}$ is an unknown finite‐dimensional parameter and $H_{k}=H_{k}\left({\bar{A}}_{k-1},{\bar{L}}_{k}\right)$ is a known vector function of $\left({\bar{A}}_{k-1},{\bar{L}}_{k}\right)$ whose first element equals 1, For example, $H_{k}={\left(1,A_{k-1},L_{k}^{⊤}\right)}^{⊤}$. The dispersion parameter $\phi_{k}$ is assumed not to depend on ${\bar{A}}_{k-1}$ or ${\bar{L}}_{k}$, and g is the canonical link function. The methods described in Sections 4.1 to 4.3 consistently estimate $\psi_{k\left(l\right)}$ when Models $M_{k}$ and $A_{k}$ (k=0,…,K) are correctly specified. Method 1 can be applied using standard GLM software. Methods 2 and 3 improve on method 1 by using more efficient estimators that are closely related to that described by DMTV in the setting of a single baseline exposure. Method 3 gives consistent estimation under slightly weaker conditions than method 2, but is more computationally intensive.

### 4.1 Method 1: Fitting the GLM implied by Models $M_{k}$ and $A_{k}$

Model $A_{k}$ states that $A_{k}$ given ${\bar{A}}_{k-1},{\bar{L}}_{k}$ and T⩾k obeys a GLM. Bayes’ rule shows (see Web Appendix A) that Models $A_{k},M_{k},\text{…},M_{K}$ and NUC together imply that for any $t⩾k,A_{k}$ given ${\bar{A}}_{k-1},{\bar{L}}_{k}$ and $T\left({\bar{A}}_{k},0\right)⩾t$ obeys the same GLM but with the intercept shifted by a function of t. Specifically, for t⩾k,

(3) $$g\left\{E\left(A_{k}|{\bar{A}}_{k-1},{\bar{L}}_{k},T\left({\bar{A}}_{k},0\right)⩾t\right)\right\}=\alpha_{k0}^{⊤}H_{k}+\alpha_{k}^{⊤}v_{k}\left(t\right),$$

where $\alpha_{k}={\left(\alpha_{k\left(k\right)},\text{…},\alpha_{k\left(K\right)}\right)}^{⊤}$ and $\alpha_{k\left(l\right)}=-\psi_{k\left(l\right)}\phi_{k}$ (l=k,…,K). Our first estimation method for $\psi_{k\left(l\right)}$ involves fitting this GLM to estimate $\alpha_{k\left(l\right)}$ and calculating $\psi_{k\left(l\right)}=-\alpha_{k\left(l\right)}∕\phi_{k}$. We now explain in more detail.

First we estimate $\psi_{k\left(k\right)}$ (k=0,…,K) as follows. For t∈[k,k+1), events $\left\{T\left({\bar{A}}_{k},0\right)⩾t\right\}$ and {T⩾t} are equivalent, and so Equation (3) implies $g\left\{E\left(A_{k}|{\bar{A}}_{k-1},{\bar{L}}_{k},T⩾t\right)\right\}=\alpha_{k0}^{⊤}H_{k}+\alpha_{k\left(k\right)}\left(t-k\right)$ for any t∈[k,k+1). Hence, a consistent estimate ${\hat{\alpha }}_{k\left(k\right)}$ of $\alpha_{k\left(k\right)}$ can be obtained as follows. For each of a number (we used 10) of equally spaced values of t between k and k+1 (including k and k+1), identify the set of individuals with T⩾t and, for each of these individuals, create a copy (a “pseudo‐individual”) with the same value of $\left({\bar{A}}_{K},{\bar{L}}_{K}\right)$ and with new random variable Q equal to t. Fit the GLM $g\left\{E\left(A_{k}|{\bar{A}}_{k-1},{\bar{L}}_{k},Q\right)\right\}=\alpha_{k0}^{⊤}H_{k}+\alpha_{k\left(k\right)}\left(Q-k\right)$ to the resulting set of (up to 10n) pseudo‐individuals. A consistent estimate of $\psi_{k\left(k\right)}$ is then ${\hat{\psi }}_{k\left(k\right)}^{\text{M1}}=-{\hat{\alpha }}_{k\left(k\right)}∕\phi_{k}$. When $\phi_{k}$ is unknown, it can be estimated by fitting Model $A_{k}$ to those of the original n individuals with T⩾k. In the simulation study of Section 7, we also tried using 50 values of t to construct the set of pseudo‐individuals instead of 10, but found this made very little difference to the estimates.

Next we estimate $\psi_{k\left(k+1\right)}$ (k=0,…,K−1). When t∈[k+1,k+2), Equation (3) is $g\left\{E\left(A_{k}|{\bar{A}}_{k-1},{\bar{L}}_{k},T\left({\bar{A}}_{k},0\right)⩾t\right)\right\}=\alpha_{k0}^{⊤}H_{k}+\alpha_{k\left(k\right)}+\alpha_{k\left(k+1\right)}\left(t-k-1\right)$. If $T_{i}\left({Ā}_{ki},0\right)$ were known for all $i,\psi_{k\left(k+1\right)}$ could be estimated just as $\psi_{k\left(k\right)}$ was, but it is not. However, as shown in Web Appendix B, $M_{k},\text{…},M_{K}$ imply that for t⩾k+1,

(4) $$P\left\{T\left({\bar{A}}_{k},0\right)⩾t|{\bar{A}}_{k},{\bar{L}}_{k},T\left({\bar{A}}_{k},0\right)⩾k\right\}=E\left\{R\left(t\right)w_{k}\left(t\right)|{\bar{A}}_{k},{\bar{L}}_{k},T⩾k\right\},$$

where $w_{k}\left(t\right)=\prod_{j=k+1}^{K}\text{exp}\left\{A_{j}v_{j}{\left(t\right)}^{⊤}\psi_{j}\right\}$. That is, within the population stratum defined by any given value of $\left({\bar{A}}_{k},{\bar{L}}_{k}\right)$ and by $T\left({\bar{A}}_{k},0\right)⩾k$ (or equivalently T⩾k), the proportion of individuals with $T\left({\bar{A}}_{k},0\right)⩾t$ is equal to the proportion of individuals with T⩾t after weighting each individual by $w_{k}\left(t\right)$. Remembering that the first element of $H_{k}$ equals one for all individuals, it follows that a consistent estimate ${\hat{\alpha }}_{k\left(k+1\right)}$ of $\alpha_{k\left(k+1\right)}$ can be obtained by fitting the GLM $g\left\{E\left(A_{k}|{\bar{A}}_{k-1},{\bar{L}}_{k},Q\right)\right\}=\alpha_{k0}^{⊤}H_{k}+\alpha_{k\left(k+1\right)}\left(Q-k-1\right)$ to a set of pseudo‐individuals constructed as described above but using 10 values of t between k+1 and k+2 (rather than k and k+1) and using weights $w_{k}\left(Q\right)=\text{exp}\left\{A_{k+1}\psi_{k+1\left(k+1\right)}\left(Q-k-1\right)\right\}$. The weights $w_{k}\left(Q\right)$ depend on $\psi_{k+1\left(k+1\right)}$, which is unknown, and so we replace it by its previously obtained estimate ${\hat{\psi }}_{k\left(k\right)}^{\text{M1}}$. A consistent estimate of $\psi_{k\left(k+1\right)}$ is then ${\hat{\psi }}_{k\left(k+1\right)}^{\text{M1}}=-{\hat{\alpha }}_{k\left(k+1\right)}∕\phi_{k}$.

In general, $\psi_{k\left(l\right)}$ (0⩽k⩽l⩽K) is estimated by ${\hat{\psi }}_{k\left(l\right)}^{\text{M1}}=-{\hat{\alpha }}_{k\left(l\right)}∕\phi_{k}$, where ${\hat{\alpha }}_{k\left(l\right)}$ is the estimate of $\alpha_{k\left(l\right)}$ obtained by fitting the GLM

(5) $$g\left\{E\left(A_{k}|{\bar{A}}_{k-1},{\bar{L}}_{k},Q\right)\right\}=\alpha_{k0}^{⊤}H_{k}+\alpha_{k\left(l\right)}\left(Q-l\right)$$

to a set of pseudo‐individuals constructed using 10 equally spaced values of t between l and l+1 and using weights $w_{k}\left(Q\right)$, with $\psi_{j\left(m\right)}$ replaced by ${\hat{\psi }}_{j\left(m\right)}^{\text{M1}}$. For later reference, we denote the fitted value of $E\left(A_{k}|{\bar{A}}_{k-1},{\bar{L}}_{k},Q=t\right)$ thus obtained as ${\hat{e}}_{k\left(l\right)}\left({\bar{A}}_{k-1},{\bar{L}}_{k},t\right)$. This is an estimate of $E\left(A_{k}|{\bar{A}}_{k-1},{\bar{L}}_{k},T\left({Ā}_{k},0\right)⩾t\right)$. Note that ${\hat{\psi }}_{j\left(m\right)}^{\text{M1}}$ (k<j⩽m⩽l) must be calculated before ${\hat{\psi }}_{k\left(l\right)}^{\text{M1}}$. If $\phi_{k}$ is unknown, it is estimated by fitting Model $A_{k}$ to the original individuals with T⩾k.

Although this estimation procedure involves weights $w_{k}\left(t\right)$, these are different from the inverse probability of exposure weights used to fit MSMs and do not suffer the same instability that can plague the latter weights. In particular, if $\psi_{k\left(k\right)}=\text{⋯}=\psi_{k\left(K\right)}=0$, that is, $A_{k}$ has no direct effect on survival, then $w_{k}\left(t\right)=1$. The variance of the weights can be reduced by using modified (or “stabilized”) weights $w_{k}^{*}\left(Q\right)$ in place of $w_{k}\left(Q\right)$, where $w_{k}^{*}\left(t\right)=\text{exp}\left\{\sum_{j=k+1}^{K}\Delta_{j\left(k\right)}^{*}v_{j}{\left(t\right)}^{⊤}\psi_{j}\right\}$ and $\Delta_{j\left(k\right)}^{*}=A_{j}-E\left(A_{j}|{\bar{A}}_{k-1},{\bar{L}}_{k},T⩾j\right)$ (j=k+1,…,K). This may improve efficiency, especially when $A_{j}$ is precisely predicted by $\left({\bar{A}}_{k-1},{\bar{L}}_{k}\right)$. The ratio $w_{k}^{*}\left(Q\right)∕w_{k}\left(Q\right)$ depends only on ${\bar{A}}_{k-1}$ and ${\bar{L}}_{k}$, and as model (5) is conditional on these, ${\hat{\alpha }}_{k\left(l\right)}$ remains consistent. Since $E\left(A_{j}|{\bar{A}}_{k-1},{\bar{L}}_{k},T⩾j\right)$ (j=k+1,…,K) is unknown, a working model $C_{j\left(k\right)}$ is specified for it and its parameters estimated from the set of individuals still at risk at time j. Note that $C_{j\left(k\right)}$ does not need to be correctly specified for ${\hat{\psi }}_{k\left(l\right)}$ to be consistent; indeed $C_{j\left(k\right)}$ need not be compatible with $A_{k}$.

### 4.2 Method 2: G‐estimation

The principle underlying the following estimator of $\psi_{k\left(l\right)}$ is that after removing the effects of $A_{k}$ and later exposures from the increment in the counting process N(t)=I(T⩾t), NUC implies that the resulting “blipped down” increment at any time t⩾k is independent of $A_{k}$ conditional on ${\bar{A}}_{k-1}$ and ${\bar{L}}_{k}$ and being still at risk.

First estimate $\psi_{k\left(k\right)}$ (k=0,…,K) by solving unbiased estimating equation

(6) $$\sum_{i=1}^{n}\int_{k}^{k+1}R_{i}\left(t\right)\Delta_{ki}\left(t\right)\left\{dN_{i}\left(t\right)-A_{ki}\;\psi_{k\left(k\right)}\;dt\right\}=0,$$

where $\Delta_{k}\left(t\right)=A_{k}-E\left(A_{k}|{\bar{A}}_{k-1},{\bar{L}}_{k},T\left({\bar{A}}_{k},0\right)⩾t\right)$. The expectation $E\left(A_{k}|{\bar{A}}_{k-1},{\bar{L}}_{k},T\left({\bar{A}}_{k},0\right)⩾t\right)$ is unknown, so we replace it by ${\hat{e}}_{k\left(k\right)}\left({\bar{A}}_{k-1},{\bar{L}}_{k},t\right)$, obtained exactly as in method 1. The next paragraph provides a rationale for Equation (6).

NUC implies that the counting process $N_{\left({\bar{A}}_{k-1},0\right)}\left(t\right)=I\left(T\left({\bar{A}}_{k-1},0\right)⩽t\right)$ for $T\left({\bar{A}}_{k-1},0\right)$ is conditionally independent of $A_{k}$ given ${\bar{A}}_{k-1},{\bar{L}}_{k}$ and $T\left({\bar{A}}_{k-1},0\right)⩾k$. We do not observe $N_{\left({\bar{A}}_{k-1},0\right)}\left(t\right)$ but Equation (2) relates $N_{\left({\bar{A}}_{k-1},0\right)}\left(t\right)$ to $N_{\left({\bar{A}}_{k},0\right)}\left(t\right)$, the counting process for $T\left({\bar{A}}_{k},0\right)$, and we do observe $N_{\left({\bar{A}}_{k},0\right)}\left(t\right)$ when t∈[k,k+1), because then it equals N(t)=I(T⩽t), the counting process for the observed failure time T. In particular, Equation (2) implies that, for any t∈[k,k+1) and conditional on $\left({\bar{A}}_{k},{\bar{L}}_{k}\right)$, the expected increment in $N_{\left({\bar{A}}_{k-1},0\right)}\left(t\right)$ during short time interval (t,t+δ] given $T\left({\bar{A}}_{k-1},0\right)⩾t$ can be unbiasedly estimated by the corresponding mean of the observed increments in N(t) minus $A_{k}\psi_{k\left(k\right)}\delta$ among the survivors at time t. Hence, the adjusted observed increment $N\left(t+\delta \right)-N\left(t\right)-A_{k}\psi_{k\left(k\right)}\delta$ should be uncorrelated with $A_{k}$ given $\left({\bar{A}}_{k-1},{\bar{L}}_{k}\right)$ and T⩾t.

DMTV derived the semiparametric efficient estimating equation for $\psi_{k\left(k\right)}$ under Model $M_{k}$ assuming known distribution of $A_{k}$ given $\left({\bar{A}}_{k-1},{\bar{L}}_{k}\right)$ and T⩾k. This equation involves inverse weighting by the hazard function; such weighting also features in efficient estimating equations of other additive hazards models. In practice, accurate estimation of the hazard function is difficult and increases the computational complexity of the procedure, and so this weighting is commonly omitted by standard fitting procedures for additive hazards models. Results of DMTV imply (see Web Appendix C) that if this is done with the semiparametric efficient equation for $\psi_{k\left(k\right)}$ under Model $M_{k}$ and if $E\left\{dN_{\left({Ā}_{k-1},0\right)}\left(t\right)|{\bar{A}}_{k},{\bar{L}}_{k},T\left({\bar{A}}_{k-1},0\right)⩾t\right\}=\gamma_{k\left(k\right)}^{⊤}H_{k}$ for all t∈[k,k+1), the result is Equation (6).

To make Equation (6) invariant to additive transformations of $A_{k}$, we replace $A_{ki}\psi_{k\left(k\right)}$ by $\Delta_{ki}\left(k\right)\psi_{k\left(k\right)}$. Since $E\left(A_{k}|{\bar{A}}_{k-1},{\bar{L}}_{k},T\left({\bar{A}}_{k},0\right)⩾k\right)$ is a constant given $\left({\bar{A}}_{k-1},{\bar{L}}_{k}\right)$, this does not affect the unbiasedness of the estimating equations. Let ${\hat{\psi }}_{k\left(k\right)}^{\text{M2}}$ denote the resulting estimator of $\psi_{k\left(k\right)}$.

Next estimate $\psi_{k\left(k+1\right)}$ using estimating equation $\sum_{i=1}^{n}\int_{k+1}^{k+2}R_{i}\left(t\right)\text{exp}\left\{A_{k+1,i}\psi_{k+1\left(k+1\right)}\left(t-k-1\right)\right\}\Delta_{ki}\left(t\right)\left[dN_{i}\left(t\right)-\left\{A_{k+1,i}\psi_{k+1\left(k+1\right)}+\Delta_{ki}\left(k+1\right)\;\psi_{k\left(k+1\right)}\right\}dt\right]=0$. The unknown $E\left(A_{k}|{\bar{A}}_{k-1},{\bar{L}}_{k},T\left({\bar{A}}_{k},0\right)⩾t\right)$ and $\psi_{k+1\left(k+1\right)}$ are replaced by ${\hat{e}}_{k\left(k+1\right)}\left({\bar{A}}_{k-1},{\bar{L}}_{k},t\right)$ and ${\hat{\psi }}_{k+1\left(k+1\right)}^{\text{M2}}$. The next paragraph provides a rationale for this estimating equation.

Again we exploit the conditional independence of $N_{\left({\bar{A}}_{k-1},0\right)}\left(t\right)$ and $A_{k}$ (NUC) and the relation between $N_{\left({\bar{A}}_{k-1},0\right)}\left(t\right)$ and $N_{\left({\bar{A}}_{k},0\right)}\left(t\right)$ but now over time interval [k+1,k+2). An added complication is that $N_{\left({\bar{A}}_{k},0\right)}\left(t\right)$ is not observed when t>k+1. However, we know from Equation (2) that when t∈[k+1,k+2) the intensities of $N_{\left({\bar{A}}_{k},0\right)}\left(t\right)$ and $N\left(t\right)=N_{\left({\bar{A}}_{k+1},0\right)}\left(t\right)$ differ by $A_{k+1}\psi_{k+1\left(k+1\right)}$ and (as noted in Section 4.1) there are $w_{k}\left(t\right)=\text{exp}\left\{A_{k+1}\psi_{k+1\left(k+1\right)}\left(t-k-1\right)\right\}$ times as many individuals with $T\left({\bar{A}}_{k},0\right)⩾t$ in the population as there are with $T\left({\bar{A}}_{k+1},0\right)⩾t$. So, we can unbiasedly estimate the expected increment in $N_{\left({\bar{A}}_{k-1},0\right)}\left(t\right)$ over small interval [t,t+δ) as the weighted mean of the increments in N(t) minus $\left(A_{k+1}\psi_{k+1\left(k+1\right)}+A_{k}\psi_{k\left(k+1\right)}\right)\delta$ with weights $\text{exp}\left\{A_{k+1}\psi_{k+1\left(k+1\right)}\left(t-k-1\right)\right\}$. This justifies the above estimating equation but with $A_{ki}\psi_{k\left(k+1\right)}$ in place of $\Delta_{ki}\left(k+1\right)\psi_{k\left(k+1\right)}$. We use $\Delta_{ki}\left(k+1\right)\psi_{k\left(k+1\right)}$ instead for the same reason that we replaced $A_{ki}\psi_{k\left(k\right)}$ by $\Delta_{ki}\left(k\right)\psi_{k\left(k\right)}$ in Equation (6).

In general, the consistent estimator ${\hat{\psi }}_{k\left(l\right)}^{\text{M2}}$ of $\psi_{k\left(l\right)}$ (l⩾k) is obtained by solving

(7) $$\begin{array}{c} \overset{n}{\underset{i=1}{\sum }}\int_{l}^{l+1}R_{i}\left(t\right)w_{ki}(t)\Delta_{ki}\;\left(t\right)\\ \times \left[dN_{i}\left(t\right)-\left\{\overset{l}{\underset{j=k+1}{\sum }}A_{ji}\psi_{j\left(l\right)}+\Delta_{ki}(l)\psi_{k\left(l\right)}\;\right\}\;dt\right]=0 \end{array}$$

with $E\left(A_{k}|{\bar{A}}_{k-1},{\bar{L}}_{k},T\left({\bar{A}}_{k},0\right)⩾t\right)$ replaced by ${\hat{e}}_{k\left(l\right)}\left({\bar{A}}_{k-1},{\bar{L}}_{k},t\right)$ and $\psi_{j\left(l\right)}$ (j>k) replaced by ${\hat{\psi }}_{j\left(l\right)}^{\text{M2}}$; this requires that $\psi_{j\left(m\right)}$ (k<j⩽m⩽l) be estimated before $\psi_{k\left(l\right)}$. The estimator ${\hat{\psi }}_{k\left(l\right)}^{\text{M2}}$ is available in closed form (see Web Appendix E for formulae when g(.) is the identity or logistic link function).

In Web Appendix F we prove ${\hat{\psi }}_{k\left(l\right)}^{\text{M2}}$ is double robust in the following sense. Let $e_{k\left(l\right)}^{*}\left({\bar{A}}_{k-1},{\bar{L}}_{k},t\right)$ denote the limit as n→∞ of ${\hat{e}}_{k\left(l\right)}\left({\bar{A}}_{k-1},{\bar{L}}_{k},t\right)$, and let Model $B_{k\left(l\right)}$ (l⩾k) be defined by the restriction $E\left\{dN_{\left({\bar{A}}_{k-1},0\right)}\left(t\right)|{\bar{A}}_{k},{\bar{L}}_{k},T\left({\bar{A}}_{k-1},0\right)⩾t\right\}=\left\{\gamma_{k\left(l\right)}^{⊤}H_{k}-e_{k\left(l\right)}^{*}\left({\bar{A}}_{k-1},{\bar{L}}_{k},k\right)\;\psi_{k\left(l\right)}\right\}dt\;\forall t\in \left[l,l+1\right),$ where $\gamma_{k\left(l\right)}$ are unknown parameters. ${\hat{\psi }}_{k\left(l\right)}^{\text{M2}}$ is consistent if (a) $M_{k},\text{…},M_{l}$, (b) either $A_{k}$ or $B_{k\left(l\right)}$, and (c) for each j=k+1,…,l, either $A_{j}$ or all of $B_{j\left(j\right)},\text{…},B_{j\left(l\right)}$ are correctly specified. The term $e_{k\left(l\right)}^{*}\left({\bar{A}}_{k-1},{\bar{L}}_{k},k\right)\psi_{k\left(l\right)}$ in Model $B_{k\left(l\right)}$ arises because of the use of $\Delta_{k}\left(l\right)\psi_{k\left(l\right)}$, rather than $A_{k}\psi_{k\left(l\right)}$, in Equation (7) (see proof). Note that if $\psi_{k\left(l\right)}=0$ or $A_{k}$ is a linear regression, so that $e_{k\left(l\right)}^{*}\left({\bar{A}}_{k-1},{\bar{L}}_{k},k\right)\;\psi_{k\left(l\right)}$ is a linear function of $H_{k}$, it can be omitted. As in method 1, efficiency may be gained by using stabilized weights $w_{ki}^{*}\left(t\right)$ in place of $w_{ki}\left(t\right)$ in Equation (7). Also, to make ${\hat{\psi }}_{k\left(l\right)}^{\text{M2}}$ invariant to additive transformations of $A_{k+1},\text{…},A_{l}$, the term $A_{ji}\psi_{j\left(l\right)}$ can be replaced by $\Delta_{j\left(k\right),i}^{*}\psi_{j\left(l\right)}$.

### 4.3 Method 3: Improved G‐estimation

If we use a different estimator ${\hat{e}}_{k\left(l\right)}\left({\bar{A}}_{k-1},{\bar{L}}_{k},t\right)$ of $E\left(A_{k}|{\bar{A}}_{k-1},{\bar{L}}_{k},T\left({\bar{A}}_{k},0\right)⩾t\right)$ for the $\Delta_{k}\left(t\right)$ and $\Delta_{k}\left(l\right)$ terms in Equation (7), then the estimator solving (7) remains consistent under a more general version of Model $B_{k\left(l\right)}$. In methods 1 and 2, ${\hat{e}}_{k\left(l\right)}\left({\bar{A}}_{k-1},{\bar{L}}_{k},t\right)$ is calculated by fitting a single GLM to a set of pseudo‐individuals, with time since lth visit, Q−l, as a covariate. In method 3, we instead fit a separate GLM at each time since the lth visit. That is, for any t⩾k, we calculate ${\hat{e}}_{k\left(l\right)}\left({\bar{A}}_{k-1},{\bar{L}}_{k},t\right)$ by fitting the GLM $g\left\{E\left(A_{k}|{\bar{A}}_{k-1},{\bar{L}}_{k}\right)\right\}=\alpha_{k0}{\left(t\right)}^{⊤}H_{k}$ to the set of individuals with T⩾t, using weights $w_{k}\left(t\right)$. This set changes only at times t at which an individual leaves the risk set, and so the GLM needs to be fitted only at these times. This is the approach taken by DMTV, who denoted the resulting estimator of $\psi_{k\left(k\right)}$ as “${\hat{\psi }}_{\text{TV PS‐DR}}$” and, on the basis of results from a simulation study, recommended it over three alternatives. As in method 2, we can use stabilized weights and replace $A_{j}\psi_{j\left(l\right)}$ by $\Delta_{j\left(k\right)}^{*}\psi_{j\left(l\right)}$. As shown in Web Appendix F, method 3 has the same double robustness property as method 2 but with the parameters $\gamma_{k\left(l\right)}$ in Model $B_{k\left(l\right)}$ now allowed to be a function of t−l.

### 4.4 Constraining exposure effects

In some applications, it may be desirable to impose the constraint that $\psi_{k\left(k+m\right)}=\psi_{k\prime \left(k\prime +m\right)}$ for all k,k′,m, that is, the effect of exposure measured at one visit k on the hazard m visits later is the same for all k. This reduces the number of parameters and, as we see in Section 7, increases the precision of their estimates. In Web Appendix G we explain how estimation may be performed under this constraint. See Vansteelandt and Sjolander (2016) for how to impose other constraints.

## 5 THE GENERAL SNCSTM

In this section, we extend the SNCSTM to allow visit times to be irregular, that is, to vary from one individual to another, and effect modification, that is, the effect of exposure on survival to depend on the exposure and confounder histories.

Let $S_{ki}$ denote the time of individual i’s kth follow‐up visit (k=1,…,K), and let $S_{0i}=0$ (i=1,…,n) and ${\bar{S}}_{i}={\left(S_{1i},\text{…},S_{Ki}\right)}^{⊤}$. Until now, we have assumed $S_{ki}=k\forall i$. We assume visit times $\bar{S}$ are planned or randomly chosen at baseline using only baseline confounder information $L_{0}$, and we modify NUC to be $T\left({\bar{A}}_{k-1},0\right)⊥\;⊥A_{k}|{\bar{L}}_{k},{\bar{A}}_{k-1},\bar{S},T⩾S_{k}\left(k=0,\text{…},K\right)$. Also, let $S_{K+1,i}$ denote an administrative censoring time common to all individuals (until now, we assumed $S_{K+1,i}=K+1$). If there is no such time, let $S_{K+1,i}=\infty$. To allow effect modification, we define $Z_{k\left(l\right)}={\left(1,Z_{k\left(l\right)}^{\mathrm{int⊤}}\right)}^{⊤}$, where $Z_{k\left(l\right)}^{\mathrm{int}}$ is a known (possibly vector) function of $\left({\bar{A}}_{k-1},{\bar{L}}_{k},\bar{S}\right)$ (“int” stands for “interactions”), and let $Z_{k}={\left(Z_{k\left(k\right)}^{⊤},\text{…},Z_{k\left(K\right)}^{⊤}\right)}^{⊤}$.

For each k=0,…,K, let $M_{k}$ be the model defined by the restriction

(8) $$\frac{P\left\{T\left({\bar{A}}_{k},0\right)⩾t|{\bar{A}}_{k},{\bar{L}}_{k},\bar{S},T⩾S_{k}\right\}}{P\left\{T\left({\bar{A}}_{k-1},0\right)⩾t|{\bar{A}}_{k},{\bar{L}}_{k},\bar{S},T⩾S_{k}\right\}}=\text{exp}\left\{-A_{k}v_{k}{\left(t,Z_{k},\bar{S}\right)}^{⊤}\psi_{k}\right\},$$

where $v_{k}\left(t,Z_{k},\bar{S}\right)$ equals ${\left(\left(t-S_{k}\right)Z_{k\left(k\right)}^{⊤},0,\text{…},0\right)}^{⊤}$ if $t\in \left[S_{k},S_{k+1}\right)$, equals ${\left(\left(S_{k+1}-S_{k}\right)Z_{k\left(k\right)}^{⊤},\left(t-S_{k+1}\right)Z_{k\left(k+1\right)}^{⊤},0,\text{…},0\right)}^{⊤}$ if $t\in \left[S_{k+1},S_{k+2}\right)$, and equals $(\left(S_{k+1}-S_{k}\right)Z_{k\left(k\right)}^{⊤},$ $\left(S_{k+2}-S_{k+1}\right)Z_{k\left(k+1\right)}^{⊤},$ ${\left(t-S_{k+2})Z_{k(k+2)}^{⊤},0,\text{…},0\right)}^{⊤}$ if $t\in \left[S_{k+2},S_{k+3}\right)$, and so forth. If $S_{k}=k$ and $Z_{k\left(l\right)}=1$, Equation (8) reduces to Equation (1). Model $M_{k}$ can also be written as $E\left\{dN_{\left({\bar{A}}_{k-1},0\right)}\left(t\right)|{\bar{A}}_{k},{\bar{L}}_{k},\bar{S},T\left({\bar{A}}_{k-1},0\right)⩾t\right\}=E\left\{dN_{\left({\bar{A}}_{k},0\right)}\left(t\right)|{\bar{A}}_{k},{\bar{L}}_{k},\bar{S},T\left({\bar{A}}_{k},0\right)⩾t\right\}-A_{k}\psi_{k\left(l\right)}^{⊤}Z_{k\left(l\right)}\;dt$ for $t\in \left[S_{l},S_{l+1}\right)$.

The modifications to methods 1 and 2 needed to fit the general SNCSTM are simple (see Web Appendix D). Modifying method 3 is simple when visit times are regular; it is possible for irregular visit times but is fiddly. In the simulation study reported in Section 7 we found little benefit from method 3 relative to method 2 when visit times were regular, and so did not implement it for irregular times.

## 6 CENSORING

We now allow for censoring before the administrative censoring time. Let $C_{i}$ and ${\tilde{T}}_{i}$ denote individual i’s censoring and failure times, respectively. Redefine $T_{i}$ and $N_{i}\left(t\right)$ as $T_{i}={\tilde{T}}_{i}\wedge C_{i}$ and $N_{i}\left(t\right)=I\left(T_{i}⩽t,\;T_{i}<C_{i}\right)$; $R_{i}\left(t\right)$ is unchanged except that $T_{i}$ has this new meaning. With these changes, methods 1 to 3 remain valid, provided two further conditions hold (Vansteelandt and Sjolander, 2016). First, the censoring hazard does not depend on the exact failure time or future exposures or confounders. That is, the counting process, $N_{C}\left(t\right)=I\left(C⩽t\right)$, for the censoring time satisfies $E\left\{dN_{C}\left(t\right)|C⩾t,{\bar{A}}_{\left\lfloor \tilde{T}\right\rfloor },{\bar{L}}_{\left\lfloor \tilde{T}\right\rfloor },\bar{S},\tilde{T}>t,\tilde{T}\right\}=\lambda \left(t,{\bar{A}}_{\left\lfloor t\right\rfloor },{\bar{L}}_{\left\lfloor t\right\rfloor },\bar{S}\right)\forall t$, where ${\bar{A}}_{\left\lfloor t\right\rfloor }$ and ${\bar{L}}_{\left\lfloor t\right\rfloor }$ are the exposure and confounder histories up to time t and $\lambda \left(t,{\bar{A}}_{\left\lfloor t\right\rfloor },{\bar{L}}_{\left\lfloor t\right\rfloor },\bar{S}\right)$ is some function only of $\left(t,{\bar{A}}_{\left\lfloor t\right\rfloor },{\bar{L}}_{\left\lfloor t\right\rfloor },\bar{S}\right)$. The second condition, which can be weakened by using censoring weights (see Web Appendix H), is that $\lambda \left(t,{\bar{A}}_{\left\lfloor t\right\rfloor },{\bar{L}}_{\left\lfloor t\right\rfloor },\bar{S}\right)=\lambda \left(t,L_{0},\bar{S}\right)$, so censoring depends only on baseline confounders.

## 7 SIMULATION STUDY

We used a simulation study to investigate the bias and efficiency of the methods. There were K+1=4 visits and two time‐dependent confounders (ie, dim$\left(L_{k}\right)=2$). These and the exposure were generated as: $L_{0}\sim N\left(\left(0,0\right),\;\sum \right),A_{0}\sim N\left(3+{\left(0.2,0.1\right)}^{⊤}L_{0},\;0.9^{2}\right),L_{k}\sim N\left(\Omega L_{k-1}+{\left(0.1,0.05\right)}^{⊤}A_{k-1},\;\sum \right)$ and $A_{k}\sim N\left(3+{\left(0.1,0.05\right)}^{⊤}L_{k},\;0.7^{2}\right)\;(k⩾1)$, where $\sum =\left[\begin{array}{cc} 0.5 & 0.2\\ 0.2 & 0.5 \end{array}\right]\text{and}\;\Omega =\left[\begin{array}{cc} 0.2 & 0.2\\ 0.1 & 0.1 \end{array}\right]$. The hazard of failure during the interval between the kth and (k+1)th visits was $0.34+{\left(0.03,0.03\right)}^{⊤}L_{k}-0.04A_{k}-0.0145A_{k-1}I\left(k⩾1\right)-0.0055A_{k-2}I\left(k⩾2\right)-0.00245A_{k-3}I\left(k=3\right)$. For this data‐generating mechanism, $M_{k}$ (k=0,…,K) is correctly specified with no effect modification (ie, $Z_{k\left(l\right)}=1$) and the true exposure effects are $\psi_{k\left(k\right)}=-0.04,\psi_{k\left(k+1\right)}=-0.01,\psi_{k\left(k+2\right)}=-0.004$ and $\psi_{k\left(k+3\right)}=-0.002$.

We considered three scenarios: two with regular and one with irregular visit times. For regular visits, $S_{ik}=k$. For irregular visits, inter‐visit times $S_{k+1,i}-S_{ki}$ were independently uniformly distributed on [0.5,1.5]. There was administrative censoring at time 4. In one of the regular visit scenarios, there was no random censoring. In the other, and in the irregular visit scenario, there was an exponentially distributed random censoring time with mean 5. For the regular visit scenario without random censoring, the expected percentages of individuals observed to fail between visits 0 and 1, 1 and 2, 2 and 3, and between visit 3 and time 4 were 20%, 14%, 11%, and 9%, respectively. For the regular and irregular visit scenarios with random censoring, these percentages were 18%, 10%, 6%, and 4%, and the corresponding expected percentages of individuals censored were 16%, 11%, 8%, and 5%. For each scenario, we generated 1000 data sets, each with n=1000 individuals. Estimation was done with and without the constraint, which is true here, that $\psi_{k\left(k+m\right)}=\psi_{k\prime \left(k\prime +m\right)}$.

Tables 1 and 2 show for the regular visit scenario without and with random censoring, respectively, the mean estimates and standard errors (SEs) for methods 1 to 3. Results for the irregular visit scenario are in Web Appendix L. We see that all the estimators are approximately unbiased, though there is some bias for $\psi_{0\left(2\right)},\psi_{0\left(3\right)}$, and $\psi_{1\left(3\right)}$, parameters for which there is relatively little information in the data. Comparing SEs, we see that methods 2 and 3 give very similar results and that these methods are more efficient than method 1. This difference in efficiency is much greater when there is random censoring (it is even greater when visit times are irregular—see Web Appendix L). This may be because method 1, unlike 2 and 3, does not distinguish between failure and censoring (or occurrence of next visit). Although methods 2 and 3 use fitted values from the same GLM that is used in method 1, the estimating equations for methods 2 and 3 involve increments dN(t), which equal one only when a failure occurs. For methods 1 and 2, coverage of 95% bootstrap confidence intervals (using 1000 bootstraps) was close to 95% (see Table 3). Coverage was not evaluated for method 3, as it is computationally intensive to bootstrap this method for 1000 simulated data sets. Imposing the constraint that $\psi_{k\left(k+m\right)}=\psi_{k\prime \left(k\prime +m\right)}$ reduced SEs, as expected.

**Table 1:** Means (×10) and SEs (×10) of parameter estimates when n=1000, visits are regular and the only censoring is administrative

Mtd	Con	$\psi_{0\left(0\right)}$	$\psi_{0\left(1\right)}$	$\psi_{0\left(2\right)}$	$\psi_{0\left(3\right)}$	$\psi_{1\left(1\right)}$	$\psi_{1\left(2\right)}$	$\psi_{1\left(3\right)}$	$\psi_{2\left(2\right)}$	$\psi_{2\left(3\right)}$	$\psi_{3\left(3\right)}$
True		0.400	0.100	0.040	0.020	0.400	0.100	0.040	0.400	0.100	0.400
Means											
1	No	0.393	0.098	0.031	0.025	0.391	0.096	0.034	0.403	0.098	0.383
2	No	0.396	0.100	0.032	0.024	0.394	0.097	0.033	0.408	0.100	0.392
3	No	0.395	0.100	0.031	0.023	0.392	0.096	0.033	0.406	0.099	0.388
P	No	0.394	0.107	0.030	0.021	0.394	0.094	0.049	0.408	0.102	0.387
1	Yes	0.386	0.096	0.032	0.024	0.386	0.096	0.032	0.386	0.096	0.386
2	Yes	0.397	0.099	0.032	0.023	0.397	0.099	0.032	0.397	0.099	0.397
3	Yes	0.395	0.098	0.032	0.023	0.395	0.098	0.032	0.395	0.098	0.395
P	Yes	0.394	0.104	0.030	0.029	0.394	0.104	0.030	0.394	0.104	0.394
SEs											
1	No	0.177	0.187	0.199	0.218	0.243	0.254	0.260	0.251	0.273	0.272
2	No	0.169	0.180	0.191	0.204	0.237	0.246	0.253	0.240	0.262	0.267
3	No	0.169	0.179	0.190	0.204	0.236	0.245	0.252	0.239	0.260	0.265
P	No	0.196	0.290	0.349	0.397	0.265	0.376	0.452	0.270	0.384	0.300
1	Yes	0.113	0.131	0.158	0.217	0.113	0.131	0.158	0.113	0.131	0.113
2	Yes	0.109	0.129	0.151	0.203	0.109	0.129	0.151	0.109	0.129	0.109
3	Yes	0.109	0.128	0.150	0.203	0.109	0.128	0.150	0.109	0.128	0.109
P	Yes	0.126	0.206	0.306	0.494	0.126	0.206	0.306	0.126	0.206	0.126

*Note*: “Mtd” is method (“P” is Picciotto *et al*.’s method—see Section 9) and “Con” is whether constraint $\psi_{k\left(k+m\right)}=\psi_{k\prime \left(k\prime +m\right)}$ is imposed.

**Table 2:** Means (×10) and SEs (×10) of parameter estimates when n=1000, visits are regular and censoring is random

Mtd	Con	$\psi_{0\left(0\right)}$	$\psi_{0\left(1\right)}$	$\psi_{0\left(2\right)}$	$\psi_{0\left(3\right)}$	$\psi_{1\left(1\right)}$	$\psi_{1\left(2\right)}$	$\psi_{1\left(3\right)}$	$\psi_{2\left(2\right)}$	$\psi_{2\left(3\right)}$	$\psi_{3\left(3\right)}$
True		0.400	0.100	0.040	0.020	0.400	0.100	0.040	0.400	0.100	0.400
Means											
1	No	0.394	0.108	0.021	0.054	0.396	0.105	0.055	0.403	0.111	0.383
1cw	No	0.396	0.102	0.020	0.054	0.393	0.096	0.054	0.408	0.097	0.383
2	No	0.396	0.104	0.036	0.033	0.399	0.096	0.038	0.411	0.098	0.393
3	No	0.396	0.103	0.036	0.033	0.396	0.095	0.038	0.407	0.096	0.385
P	No	0.397	0.117	0.024	0.050	0.399	0.095	0.078	0.405	0.117	0.390
1	Yes	0.391	0.106	0.031	0.053	0.391	0.106	0.031	0.391	0.106	0.391
1cw	Yes	0.392	0.099	0.031	0.054	0.392	0.099	0.031	0.392	0.099	0.392
2	Yes	0.398	0.099	0.037	0.032	0.398	0.099	0.037	0.398	0.099	0.398
3	Yes	0.396	0.099	0.037	0.032	0.396	0.099	0.037	0.396	0.099	0.396
P	Yes	0.395	0.108	0.035	0.051	0.395	0.108	0.035	0.395	0.108	0.395
SEs											
1	No	0.265	0.313	0.372	0.467	0.400	0.483	0.569	0.462	0.563	0.577
1cw	No	0.201	0.234	0.373	0.469	0.298	0.346	0.572	0.348	0.424	0.406
2	No	0.180	0.211	0.252	0.304	0.276	0.313	0.380	0.317	0.385	0.373
3	No	0.180	0.211	0.251	0.303	0.275	0.310	0.375	0.314	0.380	0.367
P	No	0.219	0.389	0.571	0.728	0.334	0.557	0.855	0.385	0.652	0.457
1	Yes	0.186	0.241	0.311	0.463	0.186	0.241	0.311	0.186	0.241	0.186
1cw	Yes	0.140	0.179	0.313	0.465	0.140	0.179	0.313	0.140	0.179	0.140
2	Yes	0.130	0.162	0.211	0.303	0.130	0.162	0.211	0.130	0.162	0.130
3	Yes	0.130	0.161	0.210	0.301	0.130	0.161	0.210	0.130	0.161	0.130
P	Yes	0.157	0.282	0.475	0.802	0.157	0.282	0.475	0.157	0.282	0.157

*Note*: “Mtd” is method (“1cw” is method 1 with censoring weights; ‘P’ is Picciotto *et al*.’s method—see Section 9) and ‘Con’ is whether constraint $\psi_{k\left(k+m\right)}=\psi_{k\prime \left(k\prime +m\right)}$ is imposed.

**Table 3:** Coverage (%) of 95% bootstrap confidence intervals for methods 1, 2, and 1cw (ie, method 1 with censoring weights) when *n *= 1000, visits are regular, either there is only administrative censoring or there is random censoring, and the constraint $\psi_{k\left(k+m\right)}=\psi_{k\prime \left(k\prime +m\right)}$ is not imposed

Mtd	$\psi_{0\left(0\right)}$	$\psi_{0\left(1\right)}$	$\psi_{0\left(2\right)}$	$\psi_{0\left(3\right)}$	$\psi_{1\left(1\right)}$	$\psi_{1\left(2\right)}$	$\psi_{1\left(3\right)}$	$\psi_{2\left(2\right)}$	$\psi_{2\left(3\right)}$	$\psi_{3\left(3\right)}$
No censoring										
1	96.0	96.0	95.5	94.7	94.4	95.5	96.6	95.4	95.7	94.5
2	96.5	96.4	95.4	95.7	94.9	95.6	96.5	96.0	95.8	94.7
Random censoring										
1	95.0	95.6	96.4	94.8	95.3	95.5	95.9	95.6	96.0	95.4
1cw	96.5	96.8	96.6	95.2	95.9	97.9	95.9	97.1	97.8	97.7
2	95.7	95.7	95.9	96.1	94.9	95.9	96.7	95.9	96.6	96.1

In this simulation study, censoring times are independent of exposures and confounders, and so censoring weights (Section 6) are not required for consistent estimation of the $\psi_{k\left(l\right)}$’s. However, applying method 1 with censoring weights improved its efficiency (see method 1cw in Tables 1 and 2), probably because chance associations between exposures and censoring events are reduced in the weighted sample. Coverage of bootstrap confidence intervals (Table 3) was close to 95% for most parameters, but there was overcoverage for some parameters. Using censoring weights had no effect on the efficiency of method 2.

Web Appendix L shows results for n=250 or for a shorter follow‐up time with times between visits divided by four and administrative censoring at time 1 (and so fewer failures). These are qualitatively similar to the results in Tables 1 and 2, but with the relative inefficiency of method 1 being even more marked in the scenarios with shorter follow‐up time. Web Appendix L also describes a simulation study that demonstrates the double robustness of methods 2 and 3.

## 8 ANALYSIS OF CYSTIC FIBROSIS REGISTRY DATA

The UK CF Registry records health data on nearly all people with CF in the UK at designated approximately annual visits (Taylor‐Robinson *et al*., 2018). To illustrate the use of the SNCSTM, we used data on 2386 individuals observed during 2008 to 2016 to investigate the causal effect of the drug DNase on survival. DNase has been found to have a beneficial effect on lung function, including using Registry data (Newsome *et al*., 2019), but its effect on survival has not been studied. Baseline visit was defined as an individual's first visit during 2008 to 2015, and there were up to K=8 follow‐up visits. The (irregular) visit times were defined as years after baseline visit; the median time between visits was 1.00 years (interquartile range 0.93–1.07). Individuals were defined as “treated” if they had used DNase since the previous visit and “untreated” otherwise. Individuals treated at a visit prior to their baseline visit were excluded, as were visits prior to age 18. Administrative censoring was applied at the end of 2016 and nonadministrative censoring when an individual had a transplant or had not been seen for 18 months. The percentage of treated patients increased from 14% at the baseline visit to 52% at visit 8, and most patients who began using DNase continued to use it. There were 137 deaths during follow‐up and 653 nonadministrative censorings (including 36 transplants). Of those who died, 74 (63) were treated (untreated) at the time of death. Total follow‐up was 12 380 person‐years (py), and death rates while treated and untreated were, respectively, 0.019 (74/3930) and 0.0075 (63/8450) ${\text{py}}^{-1}$. The ratio of the probabilities of surviving for one year while treated and untreated is thus 0.981∕0.9925=0.989. However, this may be due to confounding: sicker patients being more likely to receive treatment.

We estimated the effect on survival of delaying initiation of treatment by one year. To do this, we (re)defined $A_{k}$ as $A_{k}=0$ for those treated at visit k, and $A_{k}=1$ for those untreated. Now $\text{exp}\left(-\psi_{k\left(k\right)}\right)$ represents the multiplicative causal effect of intervening to start treatment at visit k rather than at visit k+1 on the probability of surviving for at least one year after visit k, among patients who survive to, and are untreated at, visit k and conditional on confounder history ${\bar{L}}_{k}$. More generally, $\text{exp}\left(-\sum_{l=k}^{k+m-1}\psi_{k\left(l\right)}\right)$ is the effect on the probability of surviving at least m years after visit k if visits are exactly annual. We imposed the constraint $\psi_{k\left(k+m\right)}=\psi_{k\prime \left(k\prime +m\right)}$. (Potential) confounders at visit k were baseline variables sex, age, and genotype class (low, high, and not assigned), and time‐varying variables ${\text{FEV}}_{1}$%, body mass index, days of IV antibiotic use, and binary indicators for four infections (*Pseudomonas aeruginosa*, *Staphylococcus aureus*, *Burkholderia cepacia complex*, and *Aspergillus*), CF‐related diabetes, smoking, and use of other mucoactive treatments and oxygen therapy. The same variables (and treatment) were included in models for the inverse probability of censoring weights.

**Figure 1:**
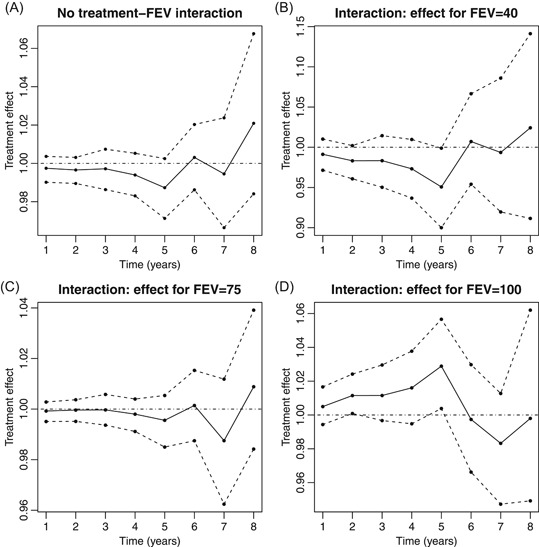
Estimates of the ratio of the survival probabilities when treatment is initiated immediately compared to initiation being delayed by one year. A: from the model with no interaction. B, C and D: from the model with interaction between treatment and ${\text{FEV}}_{1}\%$

Figure 1A shows estimates of $\text{exp}\left(-\sum_{l=k}^{k+m-1}\psi_{k\left(l\right)}\right)$ from method 2. These suggest that starting treatment now rather than waiting may cause a small decrease in the probability of survival, at least for the first 5 years: $\text{exp}\left(-\sum_{l=k}^{k+m-1}\psi_{k\left(l\right)}\right)=0.997$, 0.996, 0.997, 0.994, and 0.988 for m=1,…,5, respectively. However, the confidence intervals (obtained by bootstrapping) include 1, that is, no treatment effect. This lack of a significant treatment effect may be because we have focused on a subset of the population (adults not previously treated with DNase) and/or because there are unmeasured confounders. As expected, method 1 was very inefficient in this situation of irregular visits and substantial censoring. The confidence intervals were between 4 and 9 times wider than those from method 2.

For illustration, we also fitted an SNCSTM with an interaction between treatment and ${\text{FEV}}_{1}$%. Figure 1B to 1D shows the estimated ratios of survival probabilities for three values of ${\text{FEV}}_{1}$%: 40, 75, and 100 (the 10th, 50th, and 90th centiles of the distribution at baseline). Figure 1D suggests the ratio may actually be greater than 1 for ${\text{FEV}}_{1}$% =100, that is, starting treatment now may be better than waiting for patients with high ${\text{FEV}}_{1}$%. However, the interaction terms are not significant.

## 9 DISCUSSION

One advantage of SNCSTMs is that, in contrast to MSMs, they can cope well with situations where the inverse probabilities of exposure are highly variable. Indeed, they can even be used when the so‐called experimental treatment assignment assumption is violated, that is, when some individuals are, on the basis of their time‐varying covariate information, excluded from receiving particular exposure levels. For these individuals, $\Delta_{i}\left(t\right)=0$, meaning they do not contribute to the estimating functions of methods 1 to 3.

Another advantage of SNCSTMs is that they can be used to investigate time‐varying modification of exposure effects on survival time. Although it is, in principle, possible to do this using structural nested AFTMs, estimation difficulties caused by artificial recensoring mean that such models are usually kept simple and interactions are not explored.

The SNCSTM can also be used to estimate the counterfactual exposure‐free survivor function, that is, P{T(0)⩾t}, as $n^{-1}\sum_{i=1}^{n}R_{i}\left(t\right)\prod_{j=0}^{K}\text{exp}\left\{A_{ji}v_{j}{\left(t,Z_{ji},{\bar{S}}_{i}\right)}^{⊤}\psi_{j}\right\}$. This is because Equations (4) and (8) imply $P\left\{T\left(0\right)⩾t\right\}=E\left[R\left(t\right)\prod_{j=0}^{K}\text{exp}\left\{A_{j}v_{j}{\left(t,Z_{j},\bar{S}\right)}^{⊤}\psi_{j}\right\}\right]$. If there is censoring before time $t,R_{i}\left(t\right)$ should be inversely weighted by an estimate of $P\left(C_{i}⩾t|{\bar{A}}_{\left\lfloor t\right\rfloor i},{\bar{L}}_{\left\lfloor t\right\rfloor i},{\bar{S}}_{i}\right)$.

A limitation is that, like other additive hazards models, the SNCSTM does not constrain hazards to be nonnegative, and so does not exclude survival probabilities greater than one. Similarly, Picciotto *et al*.'s (2012) structural nested cumulative failure time model does not exclude failure probabilities greater than one.

Method 1 appears to be less efficient than methods 2 and 3 but has the appeal that it can be applied using standard GLM software. In our simulation study, the efficiency loss was fairly small when the only censoring was administrative and visit times were regular. This method became much less competitive, however, when there was random censoring, and even more so when visit times were irregular. By not distinguishing between failure and censoring, method 1 may also be more sensitive than methods 2 and 3 to violation of the assumption that $\lambda \left(t,{\bar{A}}_{\left\lfloor t\right\rfloor },{\bar{L}}_{\left\lfloor t\right\rfloor },\bar{S}\right)=\lambda \left(t,L_{0},\bar{S}\right)$. Of the three, method 3 gives consistent estimation under the weakest assumptions. However, it needs more computation than methods 1 and 2, especially when visit times are irregular and the exposure is binary. In our simulation study, methods 2 and 3 performed similarly, and so the theoretical advantage of method 3 may not be worth the extra computation. An R function for implementing our methods, with examples, is described in Web Appendix I.

DMTV discuss the close connection between their model for a point exposure (which is equivalent to the SNCSTM with K=0) and Picciotto *et al*.'s (2012) cumulative failure time model. Although the latter is a discrete‐time model for the probability of failure, it is easy to finely discretize time so as to approximate continuous time and (as Picciotto *et al*. note) to reformulate it as a model for the probability of survival. As DMTV explain, a drawback of Picciotto *et al*.'s method is the difficulty of deriving the efficient estimating equation. This difficulty arises because their class of estimating functions uses correlated survival indicators. By instead using independent increments of a counting process, DMTV were able to derive the efficient estimating function. Methods 2 and 3 are extensions to time‐varying exposures of DMTV's recommended method, and are, therefore, expected also to be more efficient than Picciotto *et al*.'s method. In Web Appendix J we elaborate on DMTV's discussion of Picciotto *et al*.'s model and reformulate it as a model for the probability of survival. Tables 1 and 2 show mean estimates and SEs for the resulting Picciotto *et al*. estimator (described in Web Appendix J and denoted “Method P” in tables). The SEs are larger than those of methods 2 and 3, suggesting methods 2 and 3 are indeed more efficient. Methods 2 and 3 also have the advantages of using closed‐form estimators, handling random censoring automatically (because estimating functions are framed in terms of increments, which are observable up to the time of censoring), and being double robust. Picciotto *et al*. use an iterative Nelder‐Mead algorithm, employ inverse probability of censoring weighting to handle random censoring, even when this censoring is completely at random, and their estimator is not double robust.

In Web Appendix K we outline how the SNCSTM can handle competing risks.

## Supporting information

Web Appendices referenced in Sections 4–7 and 9 are available with this paper at the Biometrics website on Wiley Online Library.

Supplementary Information

Supplementary Information

Supplementary Information
