# Supplementary material for: Adjusting for time‐varying confounders in survival analysis using structural nested cumulative survival time models
Source: Biometrics. 2019 Nov 7;76(2):472–83. doi: 10.1111/biom.13158 (PMC7317577; doi:10.1111/biom.13158)
Supplement: Supplementary file 2 — Supplementary Information [file BIOM-76-472-s002.pdf]

Supporting Information for 'Adjusting for Time-Varying Confounders in Survival Analysis Using Structural Nested Cumulative Survival Time Models' by Seaman, Dukes, Keogh and Vansteelandt.

The proofs, estimators and inverse probability of censoring weights in these web appendices are for the general SNCSTM described in Section 5 of the article. Proofs, estimators and weights for the simple SNCSTM with regular visit times and no effect modification are just special cases of the proofs, estimators and weights given here. Specifically, for the simple SNCSTM, $\bar{S} = (1, 2, \ldots, K)$ and $Z_{k(l)} = 1$.

In these web appendices, we write $v_k(t)^\top \psi_k$ as $G_k(t)$. Mentions of equations (1)–(8) refer to equations that appear in the article.

A. Proof of equation (3)

Model $\mathcal{A}_k$ implies we can write the probability density of A_k given $\bar{A}_{k-1}, \bar{L}_k, \bar{S}, T \geqslant S_k$ as

$$f(A_k \mid \bar{A}_{k-1}, \bar{L}_k, \bar{S}, T \geqslant S_k) = b(A_k; \phi_k) \exp\{A_k \tau - c(\tau)\}/d(\phi)$$

for some functions $b(.)$, $c(.)$ and $d(.)$ and where $\tau = \alpha_{k0}^\top H_k$ is the linear predictor. To simplify notation, we shall omit the explicit conditioning on $\bar{S}$ and instead take it as implicit.

Using Bayes' Rule, Models $\mathcal{A}_k$ and $\mathcal{M}_k$ and the no-unmeasured confounders as-

sumption, we have,

$$
\begin{aligned}
& f(A_k \mid \bar{A}_{k-1}, \bar{L}_k, T(\bar{A}_k, 0) \geqslant t) \\
& \propto f(A_k \mid \bar{A}_{k-1}, \bar{L}_k, T(\bar{A}_k, 0) \geqslant S_k) \times P(T(\bar{A}_k, 0) \geqslant t \mid \bar{A}_k, \bar{L}_k, T(\bar{A}_k, 0) \geqslant S_k) \\
& = f(A_k \mid \bar{A}_{k-1}, \bar{L}_k, T \geqslant S_k) \times P(T(\bar{A}_k, 0) \geqslant t \mid \bar{A}_k, \bar{L}_k, T(\bar{A}_k, 0) \geqslant S_k) \\
& = b(A_k; \phi_k) \exp\{A_k \tau - c(\tau)\}/d(\phi) \\
& \qquad \times P(T(\bar{A}_{k-1}, 0) \geqslant t \mid \bar{A}_k, \bar{L}_k, T(\bar{A}_{k-1}, 0) \geqslant S_k) \exp\{-A_k G_k(t)\} \\
& = b(A_k; \phi_k) \exp\{A_k \tau - c(\tau)\}/d(\phi) \\
& \qquad \times P(T(\bar{A}_{k-1}, 0) \geqslant t \mid \bar{A}_{k-1}, \bar{L}_k, T(\bar{A}_{k-1}, 0) \geqslant S_k) \exp\{-A_k G_k(t)\} \\
& \propto b(A_k; \phi_k) \exp\{A_k \tau - c(\tau)\}/d(\phi) \times \exp\{-A_k G_k(t)\} \\
& = b(A_k; \phi_k) \exp\{A_k \tau^* - c(\tau)\}/d(\phi) \\
& \propto b(A_k; \phi_k) \exp\{A_k \tau^*\}/d(\phi) \\
& \propto b(A_k; \phi_k) \exp\{A_k \tau^* - c(\tau^*)\}/d(\phi)
\end{aligned}
$$

where $\tau^* = \tau - G_k(t) d(\phi)$.

B. Proof of equation (4)

First, we prove that

$$
\begin{aligned}
& P\{T(\bar{A}_{k-1}, 0) \geqslant t \mid \bar{A}_k, \bar{L}_k, \bar{S}, T(\bar{A}_{k-1}, 0) \geqslant S_k\} \\
& \qquad = E\left[R(t) \exp\left\{ \sum_{j=k}^{K} A_j G_j(t) \right\} \mid \bar{A}_k, \bar{L}_k, \bar{S}, T \geqslant S_k \right] \qquad (9)
\end{aligned}
$$

for $t \geqslant S_k$. To simplify notation, we shall omit the explicit conditioning on $\bar{S}$ and instead take it as implicit.

For $t \geqslant S_k$,

$$
\begin{aligned}
& P\{T(\bar{A}_{k-1}, 0) \geqslant t \mid \bar{A}_k, \bar{L}_k, T(\bar{A}_{k-1}, 0) \geqslant S_k\} \\
& = P\{T(\bar{A}_k, 0) \geqslant t \mid \bar{A}_k, \bar{L}_k, T(\bar{A}_{k-1}, 0) \geqslant S_k\} \exp\{A_k G_k(t)\}
\end{aligned}
$$

Hence, for $S_k \leqslant t < S_{k+1}$,

$$P\{T(\bar{A}_{k-1},0) \geqslant t \mid \bar{A}_k, \bar{L}_k, T(\bar{A}_{k-1},0) \geqslant S_k\} \tag{10}$$

$$= P\{T \geqslant t \mid \bar{A}_k, \bar{L}_k, T \geqslant S_k\} \exp\{A_k G_k(t)\}$$

$$= E_T\{R(t) \mid \bar{A}_k, \bar{L}_k, T \geqslant S_k\} \exp\{A_k G_k(t)\}$$

$$= E_T\{R(t) \exp\{A_k G_k(t)\} \mid \bar{A}_k, \bar{L}_k, T \geqslant S_k\} \tag{11}$$

So, equation (9) has been proved for $S_k \leqslant t < S_{k+1}$.

Note that since (10) does not depend on A_k (by NUC), nor can (11). Hence, we can also write:

$$P\{T(\bar{A}_{k-1},0) \geqslant t \mid \bar{A}_{k-1}, \bar{L}_k, T(\bar{A}_{k-1},0) \geqslant S_k\} = E\left\{R(t) \exp\{A_k G_k(t)\} \mid \bar{A}_{k-1}, \bar{L}_k, T \geqslant S_k\right\} \tag{12}$$

Next, for $t \geqslant S_{k+1}$,

$$P\{T(\bar{A}_{k-1},0) \geqslant t \mid \bar{A}_k, \bar{L}_k, T(\bar{A}_{k-1},0) \geqslant S_k\}$$

$$= P\{T(\bar{A}_k,0) \geqslant t \mid \bar{A}_k, \bar{L}_k, T(\bar{A}_{k-1},0) \geqslant S_k\} \exp\{A_k G_k(t)\}$$

$$= P\{T(\bar{A}_k,0) \geqslant t \mid \bar{A}_k, \bar{L}_k, T(\bar{A}_k,0) \geqslant S_k\} \exp\{A_k G_k(t)\}$$

$$= P\{T(\bar{A}_k,0) \geqslant t \mid \bar{A}_k, \bar{L}_k, T(\bar{A}_k,0) \geqslant S_{k+1}\}$$

$$\times P\{T(\bar{A}_k,0) \geqslant S_{k+1} \mid \bar{A}_k, \bar{L}_k, T(\bar{A}_k,0) \geqslant S_k\} \exp\{A_k G_k(t)\}$$

Hence, for $S_{k+1} \leqslant t < S_{k+2}$,

$$
\begin{aligned}
&P\{T(\bar{A}_{k-1},0) \geqslant t \mid \bar{A}_{k-1}, \bar{L}_k, T(\bar{A}_{k-1},0) \geqslant S_k\} \\
&= P\{T(\bar{A}_k,0) \geqslant t \mid \bar{A}_k, \bar{L}_k, T(\bar{A}_k,0) \geqslant S_{k+1}\} \\
&\qquad \times P\{T(\bar{A}_k,0) \geqslant S_{k+1} \mid \bar{A}_k, \bar{L}_k, T(\bar{A}_k,0) \geqslant S_k\} \exp\{A_k G_k(t)\} \\
&= P\{T(\bar{A}_k,0) \geqslant t \mid \bar{A}_k, \bar{L}_k, T \geqslant S_{k+1}\} \\
&\qquad \times P\{T \geqslant S_{k+1} \mid \bar{A}_k, \bar{L}_k, T \geqslant S_k\} \exp\{A_k G_k(t)\} \\
&= E_{L_{k+1}}[P\{T(\bar{A}_k,0) \geqslant t \mid \bar{A}_k, \bar{L}_{k+1}, T \geqslant S_{k+1}\} \mid \bar{A}_k, \bar{L}_k, T \geqslant S_{k+1}] \\
&\qquad \times P\{T \geqslant S_{k+1} \mid \bar{A}_k, \bar{L}_k, T \geqslant S_k\} \exp\{A_k G_k(t)\} \\
&= E_{L_{k+1}}[E_{T,A_{k+1}}\{R(t)\exp\{A_{k+1}G_{k+1}(t)\} \mid \bar{A}_k, \bar{L}_{k+1}, T \geqslant S_{k+1}\} \mid \bar{A}_k, \bar{L}_k, T \geqslant S_{k+1}] \\
&\qquad \times P\{T \geqslant S_{k+1} \mid \bar{A}_k, \bar{L}_k, T \geqslant S_k\} \exp\{A_k G_k(t)\} \qquad \text{(using (12))} \\
&= E_{T,A_{k+1},L_{k+1}}\{R(t)\exp\{A_{k+1}G_{k+1}(t)\} \mid \bar{A}_k, \bar{L}_k, T \geqslant S_{k+1}\} \\
&\qquad \times P\{T \geqslant S_{k+1} \mid \bar{A}_k, \bar{L}_k, T \geqslant S_k\} \exp\{A_k G_k(t)\} \\
&= E_{T,A_{k+1},L_{k+1}}\{R(t)\exp\{A_k G_k(t) + A_{k+1}G_{k+1}(t)\} \mid \bar{A}_k, \bar{L}_k, T \geqslant S_{k+1}\} \\
&\qquad \times P\{T \geqslant S_{k+1} \mid \bar{A}_k, \bar{L}_k, T \geqslant S_k\} \\
&= E_{T,A_{k+1},L_{k+1}}\{R(t)\exp\{A_k G_k(t) + A_{k+1}G_{k+1}(t)\} \mid \bar{A}_k, \bar{L}_k, T \geqslant S_k\}
\end{aligned}
$$

So, equation (9) has been proved for $S_{k+1} \leqslant t < S_{k+2}$.

Using induction, the same argument can be used to prove equation (9) for $S_{k+2} \leqslant t < S_{k+3}$, then for $S_{k+3} \leqslant t < S_{k+4}$, and so on.

Now, equation (4) follows from $\mathcal{M}_k$ and equation (9), because

$$
\begin{aligned}
&P\{T(\bar{A}_k, 0) \geqslant t \mid \bar{A}_k, \bar{L}_k, T(\bar{A}_k, 0) \geqslant S_k\} \\
&= P\{T(\bar{A}_k, 0) \geqslant t \mid \bar{A}_k, \bar{L}_k, T(\bar{A}_{k-1}, 0) \geqslant S_k\} \\
&= P\{T(\bar{A}_{k-1}, 0) \geqslant t \mid \bar{A}_k, \bar{L}_k, T(\bar{A}_{k-1}, 0) \geqslant S_k\} \exp\{-A_k G_k(t)\} \\
&= E\left[R(t) \exp\left\{\sum_{j=k}^{K} A_j G_j(t)\right\} \mid \bar{A}_k, \bar{L}_k, T \geqslant S_k\right] \exp\{-A_k G_k(t)\} \\
&= E\left[R(t) \exp\left\{\sum_{j=k+1}^{K} A_j G_j(t)\right\} \mid \bar{A}_k, \bar{L}_k, T \geqslant S_k\right] \\
&= E\left\{R(t) w_k(t) \mid \bar{A}_k, \bar{L}_k, T \geqslant S_k\right\}
\end{aligned}
$$

Note that the no unmeasured confounders assumption means that the left-hand side of equation (9) cannot depend on A_k. Hence, the right-hand side cannot depend on A_k either.

C. Relation between semiparametric efficient estimating equation for $\psi_{k(k)}$ and equation (6)

In their Section 3.1, DMTV derived the semiparametric efficient estimating equation for $\psi_{k(k)}$ when the conditional distribution of A_k given $(\bar{A}_{k-1}, \bar{L}_k)$ and $T \geqslant k$ is known. This estimating equation involves inverse weighting by the hazard function. When this inverse weighting is omitted, the semiparametric efficient estimating equation becomes

$$
\sum_{i=1}^{n} \int_{k}^{k+1} R_i(t) \Delta_{ki}(t) \left\{dN_i(t) - d\Omega_{ki}(t, \bar{A}_{ki}, \bar{L}_{ki}) - A_{ki} \psi_{k(k)}\, dt\right\} = 0, \qquad (13)
$$

where $d\Omega_k(t, \bar{A}_k, \bar{L}_k) = E\{dN(t) - A_k \psi_{k(k)}\, dt \mid \bar{A}_k, \bar{L}_k, T \geqslant t\} = E\{dN_{(\bar{A}_{k-1},0)}(t) \mid \bar{A}_k, \bar{L}_k, T(\bar{A}_{k-1}, 0) \geqslant t\}$ for $t \in [k, k+1)$. In their Section 4.1, DMTV showed that if $d\Omega_k(t, \bar{A}_k, \bar{L}_k) = \gamma_{k(k)}(t-k)^{\top} H_k$ for all $t \in [k, k+1)$ for some (possibly) time-varying parameter $\gamma_{k(k)}(t-k)$ and if the term $E(A_k \mid \bar{A}_{k-1}, \bar{L}_k, T \geqslant t)$ in $\Delta_k(t) = A_k - E(A_k \mid \bar{A}_{k-1}, \bar{L}_k, T \geqslant t)$ is estimated by fitting a separate GLM at

each time t (as we do in Method 3 — see our Section 4.3), then

$$\sum_{i=1}^{n} \int_{k}^{k+1} R_i(t)\Delta_{ki}(t)\ d\Omega_{ki}(t, \bar{A}_{ki}, \bar{L}_{ki}) = 0, \tag{14}$$

and so equation (13) reduces to equation (6). This result is also shown in our Web Appendix F. In Web Appendix F, we further show that if $E(A_k \mid \bar{A}_{k-1}, \bar{L}_k, T \geqslant t)$ is instead estimated by fitting a single GLM (as we do in Method 2), then equation (14) still holds, provided that $\gamma_{k(k)}(t-k) = \gamma_{k(k)}$ does not depend on t.

D. Estimation for the general SNCSTM

The following estimation methods, which are suitable for the general SNCSTM of Section 5, generalise those described in Section 4. They reduce to those described in Section 4 when visits times are regular with $S_k = k$ and there is no effect modification (i.e. $Z_{k(l)} = 1$).

For the general SNCSTM of Section 5, which allows for irregular visit times, $\Delta_k(t)$, $\hat{e}_{k(l)}$ and $e^*_{k(l)}$ depend, in general, on the visit times $\bar{S}$, and Model $\mathcal{A}_k$ is the GLM $g\{E(A_k \mid \bar{A}_{k-1}, \bar{L}_k, \bar{S}, T \geqslant S_k)\} = \alpha_{k0}^\top H_k$.

D.1 *Method 1*

In Method 1, $\psi_{k(l)}$ is estimated by $\hat{\psi}^{\mathrm{M1}}_{k(l)} = -\hat{\alpha}_{k(l)}/\phi_k$, where $\hat{\alpha}_{k(l)}$ is the estimate of $\alpha_{k(l)}$ given by fitting GLM

$$g\{E(A_k \mid \bar{A}_{k-1}, \bar{L}_k, \bar{S}, Q)\} = \alpha_{k0}^\top H_k + \sum_{j=k}^{l-1} \alpha_{k(j)}^\top Z_{k(j)}(S_{j+1} - S_j) + \alpha_{k(l)}^\top Z_{k(l)}(Q - S_l)) \tag{15}$$

to a set of pseudo-individuals, using weights $w_k(Q)$, where $w_k(t) = \prod_{j=k+1}^{K} \exp\{A_j\ v_j(t, Z_k, \bar{S})^\top \psi_j\}$. When visit times are regular, this set is the same as in Section 3. Otherwise the rule for constructing the set is a little more complicated and is

given in Section D.4. Let $\hat{e}_{k(l)}(\bar{A}_{k-1}, \bar{L}_k, \bar{S}, t)$ denote the fitted value of $E(A_k \mid \bar{A}_{k-1}, \bar{L}_k, \bar{S}, Q = t)$.

D.2 *Method 2*

In Method 2, $\psi_{k(l)}$ $(l \geqslant k)$ is estimated as the solution, $\hat{\psi}^{\text{M2}}_{k(l)}$, to estimating equations

$$\sum_{i=1}^{n} Z_{k(l),i} \int_{S_{li}}^{S_{l+1,i}} R_i(t) w_{ki}(t)\ \Delta_{ki}(t) \\ \times \left[dN_i(t) - \left\{ \sum_{j=k+1}^{l} A_{ji} \psi_{j(l)}^{\top} Z_{j(l),i} + \Delta_{ki}(S_{li})\ \psi_{k(l)}^{\top} Z_{k(l),i} \right\} dt \right] = 0, \quad (16)$$

where $\Delta_k(t)$ is replaced by $A_k - \hat{e}_{k(l)}(\bar{A}_{k-1}, \bar{L}_k, \bar{S}, t)$. Let Model $\mathcal{B}_{k(l)}$ $(l \geqslant k)$ be defined by $E\{dN_{(\bar{A}_{k-1},0)}(t) \mid \bar{A}_k, \bar{L}_k, \bar{S}, T(\bar{A}_{k-1}, 0) \geqslant t\} = \big\{\gamma_{k(l)}^{\top} H_k - e^{*}_{k(l)}(\bar{A}_{k-1}, \bar{L}_k, \bar{S}, k)\ \psi_{k(l)}^{\top} Z_{k(l)}\big\} dt$ for all $t \in [S_l, S_{l+1})$.

As proved in Web Appendix F, estimator $\hat{\psi}^{\text{M2}}_{k(l)}$ is consistent under the conditions 1–3 stated in Section 4.2 plus the extra condition that, unless all of $\mathcal{A}_j$ $(j = k, \ldots, l)$ are correctly specified or $Z_{k(l)} = 1$, additional covariates $Z^{\text{int}}_{k(l)} * H_k$ are included in each of the GLMs of equation (15). Here, $X * Y$ denotes all pairwise interactions between X and Y.

When $\mathcal{A}_k$ is correctly specified, the true parameter values for these additional covariates $Z^{\text{int}}_{k(l)} * H_k$ are zero and they can be omitted. In the analysis of the Cystic Fibrosis registry data that allowed for an interaction between treatment and FEV$_1$%, described in Section 8, $Z^{\text{int}}_{k(l)} * H_k$ was omitted because its inclusion caused instability in the estimates of $\hat{\psi}^{\text{M2}}_{k(l)}$.

When there is no effect modification or modification depends only on L_0 (i.e. $Z_{k(l)}$'s depend at most on L_0), stabilised weights can be used and $A_{ji}\psi_{j(l)}^{\top} Z_{j(l),i}$ in equation (16) can be replaced by $\Delta^{*}_{j(k),i}\psi_{j(l)}^{\top} Z_{j(l),i}$, where $\Delta^{*}_{j(k)} = A_j - E(A_j \mid \bar{A}_{k-1}, \bar{L}_k, \bar{S}, T_i \geqslant S_j)$.

D.3 *Method 3*

Modifying Method 3 is simple when visit times are regular: the GLM of Section 4.3 is just replaced by $g\{E(A_k \mid \bar{A}_{k-1}, \bar{L}_k)\} = \alpha_{k0}(t)^\top H_k + \sum_{j=k}^{l} \alpha_{k(j)}(t)^\top Z_{k(j)}^{\text{int}}$. As with Method 2, double robustness requires $Z_{k(l)}^{\text{int}} * H_k$ be added as covariates.

D.4 *General rule for construction of the set of pseudo-individuals*

To estimate $\psi_{k(l)}$, Methods 1 and 2 involve fitting a GLM for A_k given $\bar{A}_{k-1}, \bar{L}_{k-1}$, $\bar{S}$ and Q to a set of pseudo-individuals. The rule for constructing this set when the follow-up visit times are regular and equal to $1, 2, \ldots, K$ was described in Section 4.1 of the article. Here we describe the more general rule (of which that is a special case), which can be used even when visit times are irregular.

For any $t \geqslant 0$, let $\mathcal{I}_{k(l)}(t)$ denote the set of individuals with $T \geqslant S_k + t$ and $S_l \leqslant S_k + t < S_{l+1}$, i.e. those who t units after their kth visit are still at risk and have had their lth visit but not yet their $(l+1)$th visit. Let $q_{k(l)}^{\min}$ and $q_{k(l)}^{\max}$ denote, respectively, the minimum and maximum values of t such that the set $\mathcal{I}_{k(l)}(t)$ is not empty. For each of some number (we used 10) of equally spaced values of t between $q_{k(l)}^{\min}$ and $q_{k(l)}^{\max}$ (viz. $q_{k(l)}^{\min}, q_{k(l)}^{\min} + (q_{k(l)}^{\max} - q_{k(l)}^{\min})/9, q_{k(l)}^{\min} + 2(q_{k(l)}^{\max} - q_{k(l)}^{\min})/9, \ldots, q_{k(l)}^{\max}$), take the set $\mathcal{I}_{k(l)}(t)$ and for each individual i in this set, create a pseudo-individual with $Q = S_{ki} + t$ and the same value of $(\bar{A}_K, \bar{L}_K, \bar{S})$ as individual i. Let $\mathcal{P}_{k(l)}$ denote the resulting set of (up to $10n$) pseudo-individuals.

Note that in the special case of regular visit times, $q_{k(l)}^{\min} = S_l - S_k$ and $q_{k(l)}^{\max} = S_{l+1} - S_k$ (assuming there are still individuals at risk at time S_{l+1}). Therefore, each pseudo-individual has a value of Q equal to one of S_l, $S_l + (S_{l+1} - S_l)/9$, …, S_{l+1}.

E. Closed form of estimator for Methods 2 and 3

E.1 *Method 2*

The estimator corresponding to equation (16) is

$$\hat{\psi}_{k(l)} = \left[\sum_{i=1}^{n} Z_{k(l),i} Z_{k(l),i}^{\top} \Delta_{ki}(S_{li}) \int_{S_{li}}^{S_{l+1,i}} R_i(t) w_{ki}(t)\ \Delta_{ki}(t)\ dt\right]^{-1} \times \left[\sum_{i=1}^{n} Z_{k(l),i} \int_{S_{li}}^{S_{l+1,i}} R_i(t) w_{ki}(t)\ \Delta_{ki}(t) \times \left\{ dN_i(t) - \sum_{j=k+1}^{l} A_{ji}\ \psi_{j(l)}^{\top} Z_{j(l),i}\ dt \right\}\right] \tag{17}$$

Now,

$$\begin{aligned}
&\int_{S_l}^{S_{l+1}} R(t) w_k(t)\ \Delta_k(t) \left\{ dN(t) - \sum_{j=k+1}^{l} A_j \psi_{j(l)}^{\top} Z_{j(l)} dt \right\} \\
&= R(T) I(S_l \leqslant T < S_{l+1}) w_k(T) \Delta_k(T) dN(T) \\
&\quad - \sum_{j=k+1}^{l} A_j \psi_{j(l)}^{\top} Z_{j(l)} R(S_l) w_k(S_l) \\
&\quad\quad \times \int_0^{(T \wedge S_{l+1}) - S_l} \exp\{I(l > k)\ A_l \psi_{k(l)}^{\top} Z_{k(l)}\ t\} \\
&\quad\quad\quad \times \left\{ A_k - g^{-1}\left(g[E\{A_k \mid \bar{A}_{k-1}, \bar{L}_k, \bar{S}, T(\bar{A}_k, 0) \geqslant S_l\}] + \alpha_{k(l)}^{\top} Z_{k(l)} t \right) \right\}\ dt
\end{aligned} \tag{18}$$

Note that the term $A_k - g^{-1}\left(g[E\{A_k \mid \bar{A}_{k-1}, \bar{L}_k, \bar{S}, T(\bar{A}_k, 0) \geqslant S_l\}] + \alpha_{k(l)}^{\top} Z_{k(l)} t \right)$ in equation (18) is just $\Delta_k(t)$. This is because it follows from equation (3) of the article that

$$\begin{aligned}
\Delta_k(t) &= A_k - E\{A_k \mid \bar{A}_{k-1}, \bar{L}_k, \bar{S}, T(\bar{A}_k, 0) \geqslant t\} \\
&= A_k - g^{-1}\left(g[E\{A_k \mid \bar{A}_{k-1}, \bar{L}_k, \bar{S}, T(\bar{A}_k, 0) \geqslant t\}] \right) \\
&= A_k - g^{-1}\left\{ \alpha_{k0}^{\top} H_k + \alpha_k^{\top} v_k(t) \right\} \\
&= A_k - g^{-1}\left\{ \alpha_{k0}^{\top} H_k + \alpha_k^{\top} v_k(S_l) + \alpha_{k(l)}^{\top} Z_{k(l)} t \right\} \\
&= A_k - g^{-1}\left\{ g[E\{A_k \mid \bar{A}_{k-1}, \bar{L}_k, \bar{S}, T(\bar{A}_k, 0) \geqslant S_l\}] + \alpha_{k(l)}^{\top} Z_{k(l)} t \right\}.
\end{aligned}$$

So, it is evident from equation (18) that we need to calculate integrals of the form

$$\int_0^y \exp(Bt)\left\{A_k - g^{-1}(E+Dt)\right\}\, dt \tag{19}$$

where, more specifically,

$$\begin{aligned}
y &= (T \wedge S_{l+1}) - S_l \\
B &= \begin{cases} A_l \psi_{k(l)}^\top Z_{k(l)} & \text{if} \quad l > k \\ 0 & \text{if} \quad l = k \end{cases} \\
E &= g\{E(A_k \mid \bar{A}_{k-1}, \bar{L}_k, \bar{S}, T(\bar{A}_k, 0) \geqslant S_l)\} \\
D &= \alpha_{k(l)}^\top Z_{k(l)}
\end{aligned}$$

When g is the identify link function, expression (19) becomes

$$\begin{aligned}
&\int_0^y \exp(Bt)(A_k - E - Dt)\, dt \\
&= \int_0^y \exp(Bt)\{\Delta_k(S_l) - Dt\}\, dt \\
= &\begin{cases} B^{-1}\exp(By)\{\Delta_k(S_l) - Dy\} - B^{-1}\Delta_k(S_l) + B^{-2}D\{\exp(By) - 1\} & \text{if } B \neq 0 \\ \Delta_k(S_l)y - Dy^2/2 & \text{if } B = 0 \end{cases}
\end{aligned}$$

When g is the logit link function, expression (19) becomes

$$\begin{aligned}
&\int_0^y \exp(Bt)\left\{A_k - \frac{\exp(E+Dt)}{1+\exp(E+Dt)}\right\}\, dt \\
&= \begin{cases} A_k B^{-1}\{\exp(By) - 1\} - \int_0^y \frac{\exp\{E+Ft\}}{1+\exp(E+Dt)}\, dt & \text{if } B \neq 0 \\ A_k y - \int_0^y \frac{\exp\{E+Ft\}}{1+\exp(E+Ft)}\, dt & \text{if } B = 0 \end{cases}
\end{aligned}$$

where $F = B + D$.

In the special case where $B = 0$ (and so $F = D$) and $D \neq 0$,

$$\begin{aligned}
&\int_0^y \frac{\exp(E+Ft)}{1+\exp(E+Dt)}\, dt \\
&= \int_0^y \frac{\exp(E+Dt)}{1+\exp(E+Dt)}\, dt \\
&= D^{-1}\left[\log\{1+\exp(E+Dy)\} - \log\{1+\exp(E)\}\right]
\end{aligned}$$

When $F = 0$ and $D \neq 0$,

$$\begin{aligned}
&\int_0^y \frac{\exp(E+Ft)}{1+\exp(E+Dt)}\, dt \\
&= \exp(E) \int_0^y 1 - \frac{\exp(E+Dt)}{1+\exp(E+Dt)}\, dt \\
&= y\exp(E) - \exp(E)D^{-1}\left[\log\{1+\exp(E+Dy)\} - \log\{1+\exp(E)\}\right]
\end{aligned}$$

When $D = 0$ and $F \neq 0$,

$$\int_0^y \frac{\exp(E+Ft)}{1+\exp(E+Dt)}\, dt = \frac{\exp(E)}{F\{1+\exp(E)\}}\{\exp(Fy) - 1\}$$

When $F = D = 0$,

$$\int_0^y \frac{\exp(E+Ft)}{1+\exp(E+Dt)}\, dt = \frac{\exp(E)}{1+\exp(E)} y$$

When $F \neq 0$ and $D \neq 0$, numerical integration can be used.

E.2 *Method 3*

Rewrite the estimator of expression (17) as

$$\begin{aligned}
\hat{\psi}_{k(l)} = &\left[\sum_{i=1}^n Z_{k(l),i} Z_{k(l),i}^\top \Delta_{ki}(S_{li}) \right.\\
&\left. \times \int_0^{S_{l+1,i}-S_{li}} R_i(S_{li}+t) w_{ki}(S_{li}+t)\; \Delta_{ki}(S_{li}+t)\; dt \right]^{-1} \\
&\times \left[\sum_{i=1}^n Z_{k(l),i} \int_0^{S_{l+1,i}-S_{li}} R_i(S_{li}+t) w_{ki}(S_{li}+t)\; \Delta_{ki}(S_{li}+t) \right.\\
&\left. \times \left\{ dN_i(S_{li}+t) - \sum_{j=k+1}^{l} A_{ji}\; \psi_{j(l)}^\top Z_{j(l),i}\; dt \right\}\right] \qquad (20)
\end{aligned}$$

and rewrite $w_k(S_l + t)$ for $l > k$ and $t \in [0, S_{l+1} - S_l)$ as

$$w_k(S_l+t) = \exp\left\{ \sum_{j=k+1}^{l-1} A_j \sum_{m=j}^{l-1} \psi_{j(m)}^\top Z_{j(m)}(S_{m+1} - S_m) + t \sum_{j=k+1}^{l} A_j \psi_{j(l)}^\top Z_{j(l)} \right\},$$

with $w_k(S_k + t) = 1$ and $t \in [0, S_{k+1} - S_k)$.

In Method 3, we fit a separate model for $E(A_{ki} \mid \bar{A}_{k-1,i}, \bar{L}_{ki}, \bar{S}_i, T(\bar{A}_{ki}, 0) \geqslant S_{ki} + t)$ for each value t at which the set $\mathcal{I}_k(t)$ (defined in Web Appendix D.4) changes. Henceforth we assume that visit times are regular. Then the set $\mathcal{I}_k(t)$ changes when one of the individuals fails, is censored or has their $(l+1)$th exposure at time

$S_k + t$, i.e. when $T \wedge S_{l+1} = S_k + t$. Let $e_0 = 0$ and let $\{e_1, \ldots, e_{Q_{kl}}\}$ denote the set of distinct values of $(T_i \wedge S_{l+1}) - S_l$ that are greater than or equal to zero. So, the fitted value of $E(A_{ki} \mid \bar{A}_{k-1,i}, \bar{L}_{ki}, \bar{S}_i, T(\bar{A}_{ki}, 0) \geqslant S_l + t)$ used for $\Delta_{ki}(S_l + t)$ in equation (20) is constant over each interval $t \in [e_q, e_{q+1})$ $(q = 0, \ldots, Q_{kl} - 1)$. Consequently, we can write, for $l = k$,

$$\begin{aligned}
&\int_0^{S_{l+1}-S_l} R(S_l + t) w_k(S_l + t)\, \Delta_k(S_l + t)\, dt \\
&= \sum_{q=0}^{Q_{kl}-1} R(S_l + e_q)\, \Delta_k(S_l + e_q)(e_{q+1} - e_q)
\end{aligned}$$

and for $k > l$,

$$\begin{aligned}
&\int_0^{S_{l+1}-S_l} R(S_l + t) w_k(S_l + t)\, \Delta_k(S_l + t)\, dt \\
&= \sum_{q=0}^{Q_{kl}-1} R(S_l + e_q)\, \Delta_k(S_l + e_q) \int_{e_q}^{e_{q+1}} w_k(S_l + t) dt \\
&= \sum_{q=0}^{Q_{kl}-1} R(S_l + e_q)\, \Delta_k(S_l + e_q) \\
&\quad \times \exp\left\{ \sum_{j=k+1}^{l-1} A_j \sum_{m=j}^{l-1} \psi_{j(m)}^\top Z_{j(m)} (S_{m+1} - S_m) \right\} \\
&\quad \times \int_{e_q}^{e_{q+1}} \exp\left\{ s \sum_{j=k+1}^{l} A_j \psi_{j(l)}^\top Z_{j(l)} \right\} ds \qquad (21)
\end{aligned}$$

If $\sum_{j=k+1}^{l} A_j \psi_{j(l)}^\top Z_{j(l)} \neq 0$, then expression (21) equals

$$\begin{aligned}
&\exp\left\{ \sum_{j=k+1}^{l-1} A_j \sum_{m=j}^{l-1} \psi_{j(m)}^\top Z_{j(m)} (S_{m+1} - S_m) \right\} \left\{ \sum_{j=k+1}^{l} A_j \psi_{j(l)}^\top Z_{j(l)} \right\}^{-1} \\
&\times \sum_{q=0}^{Q_{kl}-1} R(S_l + e_q)\, \Delta_k(S_l + e_q) \\
&\times \left[\exp\left\{ e_{q+1} \sum_{j=k+1}^{l} A_j \psi_{j(l)}^\top Z_{j(l)} \right\} - \exp\left\{ e_q \sum_{j=k+1}^{l} A_j \psi_{j(l)}^\top Z_{j(l)} \right\} \right]
\end{aligned}$$

On the other hand, if $\sum_{j=k+1}^{l} A_j \psi_{j(l)}^{\top} Z_{j(l)} = 0$, then expression (21) equals

$$\exp\left\{ \sum_{j=k+1}^{l-1} A_j \sum_{m=j}^{l-1} \psi_{j(m)}^{\top} Z_{j(m)} (S_{m+1} - S_m) \right\}$$
$$\times \sum_{q=0}^{Q_{kl}-1} R(S_l + e_q)\, \Delta_k(S_l + e_q)(e_{q+1} - e_q)$$

F. Proof of double robustness of Methods 2 and 3

The basic results that justify Methods 2 and 3 are equation (9) and

$$E\{dN_{(\bar{A}_{k-1},0)}(t) \mid \bar{A}_k, \bar{L}_k, \bar{S}, T(\bar{A}_{k-1},0) \geqslant t\}$$
$$= \frac{E\left[R(t) w_k(t) \left\{ dN(t) - \sum_{j=k}^{l} A_j \psi_{j(l)}^{\top} Z_{j(l)} dt \right\} \mid \bar{A}_k, \bar{L}_k, \bar{S}, T \geqslant S_k \right]}{E\left\{ R(t) w_k(t) \mid \bar{A}_k, \bar{L}_k, \bar{S}, T \geqslant S_k \right\}} \quad (22)$$

for $l \geqslant k$ and $t \in [S_l, S_{l+1})$. We show below that equation (22) is implied by equation (9) and Models $\mathcal{M}_k, \ldots, \mathcal{M}_l$. Equation (22) means that within a stratum of the population defined by $(\bar{A}_k, \bar{L}_k, \bar{S})$ and by $T(\bar{A}_{k-1}, 0) \geqslant S_k$ (or equivalently, $T \geqslant S_k$) the counterfactual hazard when $A_k, \ldots, A_K$ are set to zero is equal to the actual hazard minus $\sum_{j=k}^{l} A_j \psi_{j(l)}^{\top} Z_{j(l)}$ after weighting individuals by $w_k(t)$. Note that, since the left-hand side of equation (22) does not depend on A_k (because of the no unmeasured confounders assumption), neither can the right-hand side.

We now prove equation (22). Again, we omit the explicit conditioning on $\bar{S}$. By taking logs of both sides of equation (9) and differentiating with respect to t and multiplying both sides by minus one, we obtain

$$E\{dN_{(\bar{A}_{k-1},0)}(t) \mid \bar{A}_k, \bar{L}_k, T(\bar{A}_{k-1},0) \geqslant S_k\}$$
$$= E\left(R(t) \exp\left\{ \sum_{j=k}^{K} A_j G_j(t) \right\} \left[dN(t) - \frac{d}{dt} \left\{ \sum_{j=k}^{K} A_j G_j(t) \right\} dt \right] \mid \bar{A}_k, \bar{L}_k, T \geqslant S_k \right)$$

It follows from this and equation (9) that

$$
\begin{aligned}
&E\{dN_{(\bar{A}_{k-1},0)}(t) \mid \bar{A}_k, \bar{L}_k, T(\bar{A}_{k-1},0) \geqslant t\} \\
&= \frac{E\{dN_{(\bar{A}_{k-1},0)}(t) \mid \bar{A}_k, \bar{L}_k, T(\bar{A}_{k-1},0) \geqslant S_k\}}{P\{T(\bar{A}_{k-1},0) \geqslant t \mid \bar{A}_k, \bar{L}_k, T(\bar{A}_{k-1},0) \geqslant S_k\}} \\
&= \frac{E\left(R(t)\exp\left\{\sum_{j=k}^K A_j G_j(t)\right\}\left[dN(t) - \frac{d}{dt}\left\{\sum_{j=k}^K A_j G_j(t)\right\}dt\right] \mid \bar{A}_k, \bar{L}_k, T \geqslant S_k\right)}{E\left\{R(t)\exp\left\{\sum_{j=k}^K A_j G_j(t)\right\} \mid \bar{A}_k, \bar{L}_k, T \geqslant S_k\right\}} \\
&= \frac{E\left(R(t)\exp\left\{\sum_{j=k+1}^K A_j G_j(t)\right\}\left[dN(t) - \frac{d}{dt}\left\{\sum_{j=k}^K A_j G_j(t)\right\}dt\right] \mid \bar{A}_k, \bar{L}_k, T \geqslant S_k\right)}{E\left\{R(t)\exp\left\{\sum_{j=k+1}^K A_j G_j(t)\right\} \mid \bar{A}_k, \bar{L}_k, T \geqslant S_k\right\}} \\
&= \frac{E\left(R(t)w_k(t)\left[dN(t) - \frac{d}{dt}\left\{\sum_{j=k}^K A_j G_j(t)\right\}dt\right] \mid \bar{A}_k, \bar{L}_k, T \geqslant S_k\right)}{E\left\{R(t)w_k(t) \mid \bar{A}_k, \bar{L}_k, T \geqslant S_k\right\}}
\end{aligned}
$$

For $t \in [S_l, S_{l+1})$, $\frac{d}{dt}\left\{\sum_{j=k}^K A_j G_j(t)\right\} = \sum_{j=k}^l A_j \psi_{j(l)}^\top Z_{j(l)}$. Hence, equation (22) holds.

We now use this result to prove consistency of estimation for Method 2. Suppose that $\psi_{j(m)}$ $(k < j \leqslant m \leqslant l)$ have already been consistently estimated by Method 2 and we are now estimating $\psi_{k(l)}$. Assume that Models $\mathcal{M}_k, \ldots, \mathcal{M}_l$ are correctly specified.

First, consider the following expression and suppose that $\mathcal{A}_k$ is correctly specified.

$$
\int_{S_l}^{S_{l+1}} R(t)w_k(t)\ \Delta_k(t)\left\{dN(t) - \sum_{j=k}^{l} A_j \psi_{j(l)}^\top Z_{j(l)}\right\}dt \tag{23}
$$

This is the same as the estimating function we use — i.e. the ith element of the left-hand side of equation (7) — (apart from the $Z_{k(l)}$ term) but with $\Delta_k(S_l)$ replaced by A_k.

Now, for any $t \in [S_l, S_{l+1})$, and using equation (22), we have

$$
\begin{aligned}
&E\left[R(t)w_k(t)\ \Delta_k(t)\left\{dN(t) - \sum_{j=k}^{l} A_j \psi_{j(l)}^\top Z_{j(l)}\right\} \mid \bar{A}_k, \bar{L}_k, T \geqslant S_k\right] \\
&= \Delta_k(t)E\{dN_{(\bar{A}_{k-1},0)}(t) \mid \bar{A}_k, \bar{L}_k, T(\bar{A}_{k-1},0) \geqslant t\} \times E\{R(t)w_k(t) \mid \bar{A}_k, \bar{L}_k, T \geqslant S_k\} \\
&= \Delta_k(t)E\{dN_{(\bar{A}_{k-1},0)}(t) \mid \bar{A}_{k-1}, \bar{L}_k, T(\bar{A}_{k-1},0) \geqslant t\} \times E\{R(t)w_k(t) \mid \bar{A}_k, \bar{L}_k, T \geqslant S_k\}
\end{aligned}
$$

Now take the expectation over A_k given $\bar{A}_{k-1}, \bar{L}_k, T \geqslant S_k$. This yields

$$
\begin{aligned}
&E\left[R(t)w_k(t)\ \Delta_k(t)\left\{dN(t) - \sum_{j=k}^{l} A_j \psi_{j(l)}^{\top} Z_{j(l)}\right\} \mid \bar{A}_{k-1}, \bar{L}_k, T \geqslant S_k\right] \\
&= E\{dN_{(\bar{A}_{k-1},0)}(t) \mid \bar{A}_{k-1}, \bar{L}_k, T(\bar{A}_{k-1},0) \geqslant t\} \qquad (24) \\
&\qquad \times E[\Delta_k(t) E\{R(t)w_k(t) \mid \bar{A}_k, \bar{L}_k, T \geqslant S_k\} \mid \bar{A}_{k-1}, \bar{L}_k, T \geqslant S_k] \\
&= E\{dN_{(\bar{A}_{k-1},0)}(t) \mid \bar{A}_{k-1}, \bar{L}_k, T(\bar{A}_{k-1},0) \geqslant t\} \\
&\qquad \times E[\Delta_k(t) P\{T(\bar{A}_k,0) \geqslant t \mid \bar{A}_k, \bar{L}_k, T(\bar{A}_k,0) \geqslant S_k\} \mid \bar{A}_{k-1}, \bar{L}_k, T \geqslant S_k] \\
&\qquad (25)
\end{aligned}
$$

Line (25) follows from equation (4).

If $\mathcal{A}_k$ is correctly specified,

$$
\begin{aligned}
&E\left[\Delta_k(t) P\{T(\bar{A}_k,0) \geqslant t \mid \bar{A}_k, \bar{L}_k, T(\bar{A}_k,0) \geqslant S_k\} \mid \bar{A}_{k-1}, \bar{L}_k, T \geqslant S_k\right] \\
&\equiv E[\{A_k - E(A_k \mid \bar{A}_{k-1}, \bar{L}_k, T(\bar{A}_k,0) \geqslant t)\} \\
&\quad \times P\{T(\bar{A}_k,0) \geqslant t \mid \bar{A}_k, \bar{L}_k, T(\bar{A}_k,0) \geqslant S_k\} \mid \bar{A}_{k-1}, \bar{L}_k, T \geqslant S_k] \\
&= P\{T(\bar{A}_k,0) \geqslant t \mid \bar{A}_{k-1}, \bar{L}_k, T(\bar{A}_k,0) \geqslant S_k\} \\
&\quad \times \{E(A_k \mid \bar{A}_{k-1}, \bar{L}_k, T(\bar{A}_k,0) \geqslant t) - E(A_k \mid \bar{A}_{k-1}, \bar{L}_k, T(\bar{A}_k,0) \geqslant t)\} \qquad (26) \\
&= 0 \qquad (27)
\end{aligned}
$$

Equation (26) follows because, for a general random variable X and a general event A, $E\{X\ P(A \mid X)\} = P(A)E(X \mid A)$. In conclusion, we have shown that equation (23) has expectation zero. Now, if we replace A_k in equation (23) by $\Delta_i(S_l)$, then we are simply adding a function of $(\bar{A}_{k-1}, \bar{L}_k)$ multiplied by $R(t)w_k(t)\Delta_k(t)$. It is straightforward to show that this extra term has expectation zero when $\mathcal{A}_k$ is

correctly specified. This is because

$$
\begin{aligned}
&E\{R(t)w_k(t)\Delta_k(t) \mid \bar{A}_k, \bar{L}_k, T(\bar{A}_k, 0) \geqslant S_k\} \\
&= \Delta_k(t)\ E\{R(t)w_k(t) \mid \bar{A}_k, \bar{L}_k, T(\bar{A}_k, 0) \geqslant S_k\} \\
&= \Delta_k(t)\ P\{T(\bar{A}_k, 0) \geqslant t \mid \bar{A}_k, \bar{L}_k, T(\bar{A}_k, 0) \geqslant S_k\}
\end{aligned}
$$

using equation (4). Now, taking the conditional expectation of this over A_k given $(A_{k-1}, \bar{L}_k)$ and $T(\bar{A}_k, 0) \geqslant S_k$, we obtain

$$
\begin{aligned}
&E\{R(t)w_k(t)\Delta_k(t) \mid \bar{A}_{k-1}, \bar{L}_k, T(\bar{A}_k, 0) \geqslant S_k\} \\
&= E\left[\Delta_k(t) P\{T(\bar{A}_k, 0) \geqslant t \mid \bar{A}_k, \bar{L}_k, T(\bar{A}_k, 0) \geqslant S_k\} \mid \bar{A}_{k-1}, \bar{L}_k, T \geqslant S_k\right],
\end{aligned}
$$

which we know, from equation (27), equals zero.

Likewise, when the $Z_{k(l)}$'s are functions only of L_0, changing A_j to $\Delta^{\dagger}_{j(k)} = A_j - E^{\dagger}(A_j \mid \bar{A}_{k-1}, \bar{L}_k, \bar{S}, T \geqslant S_k)$ $(j = k+1, \ldots, l)$ in the estimating function (23), where $E^{\dagger}(A_j \mid \bar{A}_{k-1}, \bar{L}_k, \bar{S}, T \geqslant S_k)$ denotes the limiting fitted value from Model $\mathcal{C}_{j(k)}$, simply adds a function of $(\bar{A}_{k-1}, \bar{L}_k)$ multiplied by $R(t)w_k(t)\Delta_k(t)$, and so the same is true. Finally, if the $Z_{k(l)}$'s are functions only of L_0 and we replace $w_k(t)$ by $w^*_k(t)$, we are simply multiplying the estimating function (23) by a function of $(\bar{A}_{k-1}, \bar{L}_k)$, and so it still has conditional expectation zero given $(\bar{A}_{k-1}, \bar{L}_k)$ and $T(\bar{A}_k, 0) \geqslant S_k$.

Now suppose that $\mathcal{A}_k$ may not be correctly specified but $\mathcal{B}_{k(l)}$ is correctly specified. Consider the following estimating equations for $\psi_{k(l)}$:

$$
\begin{aligned}
&\sum_{i=1}^{n} Z_{k(l),i} \int_{S_{li}}^{S_{l+1,i}} R_i(t) w_{ki}(t)\ \Delta_{ki}(t) \\
&\quad \times \left[dN_i(t) - \left\{ \gamma_{k(l)}^{\top}\ h_k(\bar{A}_{k-1,i}, \bar{L}_{ki}, \bar{S}_i) + \sum_{j=k+1}^{l} A_{ji}\psi_{j(l)}^{\top} Z_{j(l),i} + \Delta_{ki}(S_{li})\psi_{k(l)}^{\top} Z_{k(l),i} \right\} dt \right] \\
&\quad = 0
\end{aligned}
\tag{28}
$$

for any given value of $\gamma_{k(l)}$, and with $\Delta_k(t)$ replaced by its estimate obtained

as described in Section 4.2 of the article. These are the same as the estimating equations we use (i.e. equation (16)) except that they include the extra term $\gamma_{k(l)}^{\top} H_k$.

For any $t \in [S_l, S_{l+1})$, we have, using equation (22)

$$\begin{aligned}
&E\Big(R(t)w_k(t)\,\Delta_k(t)\,\big[dN(t) - \big\{\gamma_{k(l)}^{\top}\, h_k(\bar{A}_{k-1}, \bar{L}_k, \bar{S}) \\
&\qquad + \sum_{j=k+1}^{l} A_j \psi_{j(l)}^{\top} Z_{j(l)} + \Delta_k(S_l)\psi_{k(l)}^{\top} Z_{k(l)}\Big\}\, dt\Big] \mid \bar{A}_k, \bar{L}_k, T(\bar{A}_{k-1}, 0) \geqslant S_k\Big) \\
&= \big[E\{dN_{(\bar{A}_{k-1},0)}(t) \mid \bar{A}_k, \bar{L}_k, T(\bar{A}_{k-1}, 0) \geqslant t\} - \gamma_{k(l)}^{\top}\, h_k(\bar{A}_{k-1}, \bar{L}_k, \bar{S}) \\
&\quad + E\{A_k \mid \bar{A}_{k-1}, \bar{L}_k, T(\bar{A}_{k-1}, 0) \geqslant S_k\}\psi_{k(l)}^{\top} Z_{k(l)}\big] \\
&\quad \times E\{R(t)w_k(t) \mid \bar{A}_k, \bar{L}_k, T(\bar{A}_{k-1}, 0) \geqslant S_k\} \times \Delta_k(t)
\end{aligned}$$

which equals zero when Model $\mathcal{B}_{k(l)}$ holds. Hence, equations (28) are unbiased estimating equations for $\psi_{k(l)}$ when $\mathcal{B}_{k(l)}$ is correctly specified and $\gamma_{k(l)}$ equals its true value.

Finally, we shall show that, when either $Z_{k(l)} = 1$ or $Z_{k(l)}^{\text{int}} * H_k$ is included in GLM (15), then, because of the way that $E(A_{ki} \mid \bar{A}_{k-1,i}, \bar{L}_{ki}, T_i(\bar{A}_k, 0) \geqslant t)$ in $\Delta_{ki}(t)$ is estimated, we have

$$\sum_{i=1}^{n} Z_{k(l),i} \int_{S_{li}}^{S_{l+1,i}} R_i(t) w_{ki}(t)\, \Delta_{ki}(t) \times \gamma_{k(l)}^{\top} H_k\, dt = 0 \qquad (29)$$

for any value of $\gamma_{k(l)}$, regardless of whether Model $\mathcal{A}_k$ or Model $\mathcal{B}_{k(l)}$ is correctly specified. This means that equations (28) reduce to equations (16), and thus our estimating equations (16) have expectation zero when either Model $\mathcal{A}_k$ or Model $\mathcal{B}_{k(l)}$ is correctly specified.

The reason why equation (29) holds is as follows. Let X denote any element of the

vector $(H_k^\top, Z_{k(l)}^{\text{int}} * H_k^\top)^\top$. Then

$$\begin{aligned}
&\sum_{i=1}^{n}\int_{S_{li}}^{S_{l+1,i}} R_i(t)w_{ki}(t)\ \Delta_{ki}(t)\ X_i\ dt \\
&= \sum_{i=1}^{n}\int_{S_{li}-S_{ki}}^{S_{l+1,i}-S_{ki}} R_i(S_{ki}+t)w_{ki}(S_{ki}+t)\ \Delta_{ki}(S_{ki}+t)\ X_i\ dt \\
&= \sum_{i=1}^{n}\int_{0}^{\infty} I(S_{li}\leqslant S_{ki}+t<S_{k+1,i})R_i(S_{ki}+t)w_{ki}(S_{ki}+t)\ \Delta_{ki}(S_{ki}+t)\ X_i\ dt \\
&= \lim_{\delta\to\infty} \sum_{t\in\left\{q_{k(l)}^{\min},\ q_{k(l)}^{\min}+(q_{k(l)}^{\max}-q_{k(l)}^{\min})/\delta,\ q_{k(l)}^{\min}+2(q_{k(l)}^{\max}-q_{k(l)}^{\min})/\delta,\ldots,\ q_{k(l)}^{\max}\right\}} \sum_{i=1}^{n} I(S_{li}\leqslant S_{ki}+t<S_{k+1,i}) \\
&\qquad \times R_i(S_{ki}+t)w_{ki}(S_{ki}+t)\ \Delta_{ki}(S_{ki}+t)\ X_i \times \frac{q_{k(l)}^{\max}-q_{k(l)}^{\min}}{\delta} \\
&\approx \sum_{t\in\left\{q_{k(l)}^{\min},\ q_{k(l)}^{\min}+(q_{k(l)}^{\max}-q_{k(l)}^{\min})/9,\ q_{k(l)}^{\min}+2(q_{k(l)}^{\max}-q_{k(l)}^{\min})/9,\ldots,\ q_{k(l)}^{\max}\right\}} \sum_{i=1}^{n} I(S_{li}\leqslant S_{ki}+t<S_{k+1,i}) \\
&\qquad \times R_i(S_{ki}+t)w_{ki}(S_{ki}+t)\ \Delta_{ki}(S_{ki}+t)\ X_i \times \frac{q_{k(l)}^{\max}-q_{k(l)}^{\min}}{9}. \qquad (30)
\end{aligned}$$

Expression (30) equals zero because it is one element of the score function vector (multiplied by $(q_{k(l)}^{\max}-q_{k(l)}^{\min})/9$) for the GLM of equation (15) fitted to the set $\mathcal{P}_{k(l)}$ with weights $w_k(Q)$. (The set $\mathcal{P}_{k(l)}$ was defined in Web Appendix D.4.)

The proof of double robustness of Method 3 is similar. The difference lies in the way that fitted values of $E(A_{ki} \mid \bar{A}_{k-1,i}, \bar{L}_{ki}, \bar{S}_i, T_i(\bar{A}_{ki}, 0) \geqslant t)$ are estimated. Suppose for simplicity that $Z_{k(l)} = 1$ and visit times are regular. For Method 3, the score equations corresponding to GLM $g\{E(A_k \mid \bar{A}_{k-1}, \bar{L}_k\} = \alpha_{k0}(t)^\top H_k$ fitted to $\mathcal{I}_k(t)$ using weights $w_k(S_k + t)$ are

$$\sum_{i=1}^{n} R_i(S_{ki}+t)I(S_l \leqslant S_k+t<S_{l+1})w_{ki}(S_k+t)\Delta_{ki}(S_k+t)H_{ki} = 0 \qquad (31)$$

(The set $\mathcal{I}_k(t)$ was defined in Web Appendix D.4.) Hence, equation (31) holds for

any $t \geqslant 0$. Consequently, when Method 3 is used,

$$\begin{aligned}
&\sum_{i=1}^{n} \int_{S_l}^{S_{l+1}} R_i(t) w_{ki}(t)\, \Delta_{ki}(t)\, \gamma_{k(l)}(t - S_{li})^\top H_{ki}\, dt \\
&= \sum_{i=1}^{n} \int_0^\infty R_i(S_{ki} + t) I(S_l \leqslant S_k + t < S_{l+1}) w_{ki}(S_k + t)\, \Delta_{ki}(S_k + t) \\
&\qquad \times \gamma_{k(l)}(t + S_k - S_l)^\top H_{ki}\, dt \\
&= \gamma_{k(l)}(t + S_k - S_l)^\top \int_0^\infty \sum_{i=1}^{n} R_i(S_{ki} + t) I(S_l \leqslant S_k + t < S_{l+1}) \\
&\qquad \times w_{ki}(S_k + t)\, \Delta_{ki}(S_k + t) H_{ki}\, dt \\
&= 0.
\end{aligned}$$

G. Constraining exposure effects

In this web appendix we explain how estimation of parameters can be performed under the constraint that $\psi_{k(k+m)} = \psi_{k'(k'+m)}$ for all k, k', m.

For Method 1, a simple way to estimate $\psi_{k(k+m)}$ under this constraint is to calculate $\hat{\psi}^{\mathrm{M1}}_{k(k+m)}$ $(k = 0, \ldots, K - m)$ separately as before and then, for each element of the vector $\hat{\psi}^{\mathrm{M1}}_{k(k+m)}$, calculate a weighted average of these $K - m + 1$ estimates. Suitable weights are the reciprocals of the corresponding $K - m + 1$ variances estimated using a sandwich variance estimator that accounts for duplication of individuals as pseudo-individuals. It can be calculated using standard software and is a valid estimator of the variance of $\hat{\psi}^{\mathrm{M1}}_{k(k)}$, but not of $\hat{\psi}^{\mathrm{M1}}_{k(k+m)}$ when $m > 0$, because it ignores the uncertainty in $w_k(t)$ arising from estimating the ψ's. Nonetheless, it suffices for the purpose of averaging the $K - m + 1$ estimates of $\psi_{k(k+m)}$. For Methods 2 and 3, we simply sum the $K - m + 1$ estimating equations (7) for $\psi_{k(k+m)}$ $(k = 0, \ldots, K - m)$ and solve the resulting single equation.

In Section 3, we assumed the regular visit times are $0, 1, \ldots, K$, rescaling the time variable if necessary. Such rescaling may make it more reasonable to constrain

$\psi_{k(k+m)}$ to be a known multiple of $\psi_{k'(k'+m)}$. For example, if visits 1 and 2 are one and 13 months, respectively, after baseline, then $\psi_{0(0)}$ and $\psi_{1(1)}$ are measured in units of per-month and per-year, respectively. Since $\psi_{0(0)}$ and $\psi_{1(1)}/12$ are measured in the same units (per-month), one might constrain $\psi_{0(0)} = \psi_{1(1)}/12$. This requires only minor modification of the above procedures. However, using the more general SNCSTM described in Section 5 avoids the need to rescale time.

We have only considered one form of constraint on the exposure effects; Vansteelandt and Sjolander (2016) show how to impose other forms.

H. Inverse probability of censoring weighting

Assume that the first condition in Section 6 holds, namely, that

$$E\{dN_C(t) \mid C \geqslant t, \bar{A}_{\lfloor \tilde{T} \rfloor}, \bar{L}_{\lfloor \tilde{T} \rfloor}, \bar{S}, \tilde{T} > t, \tilde{T}\} = \lambda(t, \bar{A}_{\lfloor t \rfloor}, \bar{L}_{\lfloor t \rfloor}, \bar{S}). \tag{32}$$

Let $w_k^C(t) = \exp\left\{\int_{S_k}^t \lambda(s, \bar{A}_{\lfloor s \rfloor}, \bar{L}_{\lfloor s \rfloor}, \bar{S})\, ds\right\}$. This is the inverse probability of remaining uncensored at time t. A parametric model for $\lambda(t, \bar{A}_{\lfloor t \rfloor}, \bar{L}_{\lfloor t \rfloor}, \bar{S})$ is specified and its parameters (and hence $w_k^C(t)$) are estimated from the data. Now Methods 1–3 can be used with the weights $w_k(t)$ replaced by $w_k(t) \times w_k^C(t)$. If the assumptions sufficient for consistency in the absence of censoring (see Section 4) are satisfied, and equation (32) holds, and the model for $\lambda(t, \bar{A}_{\lfloor t \rfloor}, \bar{L}_{\lfloor t \rfloor}, \bar{S})$ is correctly specified, then the resulting estimates of $\psi_{k(l)}$ are consistent.

More stable weights can be obtained by specifying, for each $k = 0, \ldots, K$, an additional parametric model for $\lambda_k(s, \bar{A}_{k-1}, \bar{L}_k, \bar{S}) = E\{dN_C(t) \mid C \geqslant t, \bar{A}_{k-1}, \bar{L}_k, \bar{S}, \tilde{T} > t\}$ $(t > S_k)$. This differs from the previous model in that it is conditional only on $(\bar{A}_{k-1}, \bar{L}_k)$. After estimating the parameters of this model, $w_k^C(t)$ is replaced by $w_k^{CS}(t) = \exp\left\{\int_{S_k}^t \lambda(s, \bar{A}_{\lfloor s \rfloor}, \bar{L}_{\lfloor s \rfloor}, \bar{S})\, ds \; - \int_{S_k}^t \lambda_k(s, \bar{A}_{k-1}, \bar{L}_k, \bar{S})\, ds\right\}$. Note that misspecification of this additional model does not affect consistency of the estima-

tor of $\psi_{k(l)}$, and that if $\lambda(t, \bar{A}_{\lfloor t \rfloor}, \bar{L}_{\lfloor t \rfloor}, \bar{S}) = \lambda(t, \bar{A}_{k-1}, \bar{L}_k, \bar{S})$ for all $t \in [S_k, S_{l+1})$, then $w_k^{CS}(t) = 1$ for $t \in [S_k, S_{l+1})$, i.e. no censoring weights are needed when estimating $\psi_{k(l)}$.

The formulae given in Web Appendix E for Method 2 are easily extended to handle inverse probability of censoring weighting, provided that the parametric models for censoring are proportional hazards models with a constant baseline hazard between visits. We now explain how this is done.

Above, we referred to two parametric models, one for $\lambda(t, \bar{A}_{\lfloor t \rfloor}, \bar{L}_{\lfloor t \rfloor}, \bar{S})$ and an additional one for $\lambda_k(t, \bar{A}_{k-1}, \bar{L}_k, \bar{S})$, and said that the stabilised inverse probability of censoring weights are estimates of

$$w^{CS}(t) = \exp\left\{ \int_{S_{ki}}^{t} \lambda(s, \bar{A}_{\lfloor s \rfloor, i}, \bar{L}_{\lfloor s \rfloor, i}, \bar{S}_i)\, ds - \int_{S_{ki}}^{t} \lambda_k(s, \bar{A}_{k-1,i}, \bar{L}_{ki}, \bar{S}_i)\, ds \right\}.$$

(If unstabilised weights are used, $\lambda_k(s, \bar{A}_{k-1}, \bar{L}_k, \bar{S})$ is simply replaced by zero.)

For the first parametric model, we assume

$$\lambda(t, \bar{A}_{\lfloor t \rfloor}, \bar{L}_{\lfloor t \rfloor}, \bar{S}) = \exp\{\beta_k^\top\, b_k(\bar{A}_k, \bar{L}_k, \bar{S})\}$$

for $t \in [S_k, S_{k+1})$, where $b_k(\bar{A}_k, \bar{L}_k, \bar{S})$ is a known vector function of $(\bar{A}_k, \bar{L}_k, \bar{S})$ whose first element equals one (this is an intercept term), and β_k is an unknown vector parameter. For the second parametric model, we assume

$$\lambda_k(t, \bar{A}_{k-1}, \bar{L}_k, \bar{S}) = \exp\{\beta_{k(l)}^\top\, b_{k(l)}(\bar{A}_{k-1}, \bar{L}_k, \bar{S})\}$$

for $t \in [S_l, S_{l+1})$, where $b_{k(l)}(\bar{A}_{k-1}, \bar{L}_k, \bar{S})$ is a known vector function of $(\bar{A}_{k-1}, \bar{L}_k, \bar{S})$ whose first element equals one, and $\beta_{k(l)}$ is an unknown vector parameter. Abbreviate $b_k(\bar{A}_k, \bar{L}_k, \bar{S})$ as b_k and $b_{k(l)}(\bar{A}_{k-1}, \bar{L}_k, \bar{S})$ as $b_{k(l)}$. The inverse probability of censoring weight at time $t \in [S_l, S_{l+1})$ when estimating $\psi_{k(l)}$ is now

$$\begin{aligned} w_{k(l)}^{CS}(t) &= \prod_{j=k}^{l-1} \exp\left\{ \left(\beta_j^\top b_j - \beta_{k(j)}^\top b_{k(j)}\right) \times (S_{j+1} - S_j) \right\} \\ &\quad \times \exp\left\{ \left(\beta_l^\top b_l - \beta_{k(l)}^\top b_{k(j)}\right) \times (t - S_l) \right\} \end{aligned}$$

In particular,

$$w^{CS}_{k(l)}(S_l) = \prod_{j=k}^{l-1} \exp\left\{\left(\beta_j^\top b_j - \beta_{k(j)}^\top b_{k(j)}\right) \times (S_{j+1} - S_j)\right\}.$$

So, in equation (18), we should replace $w_k(T)$ by $w_k(T) \times w^{CS}_{k(l)}(T)$, replace $w_k(S_l)$ by $w_k(S_l) \times w^{CS}_{k(l)}(S_l)$, and replace $\exp\{I(l > k)\ A_l \psi_{k(l)}^\top Z_{k(l)}\ t\}$ by

$$\exp\left[\left\{I(l > k)\ A_l \psi_{k(l)}^\top Z_{k(l)} + (\beta_l^\top b_l - \beta_{k(l)}^\top b_{k(l)})\right\}\ t\right].$$

Similar modifications can be made to Method 3.

I. Software

Our R function *sncstm* can be used to apply Methods 1–3. Note that Method 3 is only implemented for regular visits and without inverse probability of censoring weights.

Two examples of the use of *sncstm* are provided in the files 'example1.r' and 'example2.r'.

The compulsory arguments of *sncstm* are as follows:

data : A data frame containing the following elements (here n is the number of individuals, $K+1$ is the number of visits, and p is the number of variables that are confounders in at least one of Models $\mathcal{A}_0, \ldots, \mathcal{A}_K$):

- tim — n-vector containing the failure or censoring time for each individual
- fail — n-vector containing the failure/censoring indicator for each individual (equals TRUE if fails and FALSE if censored)
- tau — $n \times (K+1)$ matrix whose $(i, k+1)$th entry is S_{ki}, the kth visit time for individual i (note that all the entries in the first column should equal zero, because $S_{0i} = 0$)

- A — $n \times (K+1)$ matrix whose ith row equals $(A_{0i}, A_{1i}, \ldots, A_{Ki})$, the treatments for individual i
- L — $n \times p$ matrix whose ith row contains the values for individual i of the p variables that are confounders in at least one of Models $\mathcal{A}_0, \ldots, \mathcal{A}_K$
- Z (optional) — if the optional argument useZ (see below) is not specified, then Z should not be specified either, but if useZ is specified, then Z should be a matrix; see Example 2 for details of how to specify Z when useZ is specified

useA : $(K+1)\times(K+1)$ matrix whose $(k+1)$th row indicates which of $A_0, A_1, \ldots, A_{k-1}$ to include as covariates in Model $\mathcal{A}_k$. If the $(k+1, j+1)$th element of useA equals TRUE, A_j is included in $\mathcal{A}_k$. If this element of useA equals FALSE, A_j is not included.

useL : $(K+1)\times p$ matrix whose $(k+1)$th row indicates which of the p confounders to include as covariates in Model $\mathcal{A}_k$. If the $(k+1, j+1)$th element of useL equals TRUE, the jth of the p confounders is included in $\mathcal{A}_k$. If this element of useL equals FALSE, the jth confounder is not included.

EXPOSETYPE : 'gaussian' if Model $\mathcal{A}_k$ is a linear regression; 'binomial' if $\mathcal{A}_k$ is a logistic regression.

The optional arguments of the *sncstm* function are as follows ('by default' means if the argument is not specified):

METHOD : Indicates which of the three estimation methods described in our article should be used to estimate the $\psi_{k(l)}$ parameters. By default, this equals 2, meaning that Method 2 is used. To use one of the other methods, set METHOD equal to 1 or 3.

CONSTRAIN : By default, this equals FALSE. If it equals TRUE, the constraint that $\psi_{k(k+m)} = \psi_{k'(k'+m)}$ for all k, k', m is imposed.

NQUAD : When visit times are regular, this is the number of equally spaced values of t between S_k and S_{k+1} at which a pseudo-individual is created from each individual still at risk. When visit times are irregular, it is the number of equally spaced values of t between $q^{\min}_{k(l)}$ and $q^{\max}_{k(l)}$ (see Web Appendix D.4). By default, this equals 10, which is the number used for the main simulation study reported in our article. If the probability of failure between consecutive visits is large (e.g. $> 10\%$ of individuals still at risk at visit k fail before visit $k+1$) or if visit times are highly irregular (so that $q^{\max}_{k(l)} - q^{\min}_{k(l)}$ is very large), it may be desirable to increase NQUAD. The aim should be to choose NQUAD to be large enough such that any further increase in NQUAD has little impact on the estimates of $\psi_{k(l)}$. The NQUAD argument is ignored if METHOD=3.

STABILISE : By default, this equals FALSE, meaning that unstabilised weights $w_k(t)$ are used. When useZ (see below) is not specified (so that the SNCSTM assumes there is no effect modification), STABILISE can be set equal to TRUE, meaning that stabilised weights $w^*_k(t)$ are used instead of $w_k(t)$.

IPCW : By default, this equals FALSE, meaning there is no inverse probability of censoring weighting. Specify IPCW=TRUE to use inverse probability of censoring weighting.

useAcensor : If IPCW=TRUE, useAcensor is a $(K+1) \times (K+1)$ matrix, whose $(k+1, j+1)$th entry equals TRUE if A_j is included in the model for the hazard of censoring during time interval $[S_k, S_{k+1})$, and equals FALSE if it is not included. Ignored if IPCW=FALSE.

useLcensor : If IPCW=TRUE, useLcensor is a $(K+1) \times p$ matrix, whose $(k+1, j+1)$th entry equals TRUE if the jth variable in the L component of the

data frame called 'data' (see above) is included in the model for the hazard of censoring during time interval $[S_k, S_{k+1})$, and equals FALSE if this variable is not included. Ignored if IPCW=FALSE.

admincens : The censoring model (i.e. the model used to calculate the inverse probability of censoring weights) considers censoring as the 'event' of interest and considers failure as a 'censoring'. By default, the censoring model treats all censorings as 'events'. However, one might not want inverse probability of censoring weights to adjust for administrative censorings. If so, adminstrative censorings need to be treated as 'censorings' rather than 'events' in the censoring model. To treat some censorings as 'censorings' rather than 'events' in the censoring model, specify admincens as a vector of length n whose ith entry equals TRUE if the censoring of individual i is to be treated by the censoring model as a 'censoring' and equals FALSE if it is to be treated as an 'event'. This argument is ignored if IPCW is not specified or if IPCW is specified to equal FALSE.

useZ : By default, there is assumed to be no effect modification, i.e. it assumes that the causal effect of A_k does not depend on the treatment or confounder histories $(\bar{A}_{k-1}, \bar{L}_k)$. This assumption corresponds to $Z_{k(l)} = 1$ in the SNCSTM. useZ can be used to indicate that the SNCSTM should instead allow the causal effect of A_k to depend on $(\bar{A}_{k-1}, \bar{L}_k)$. In that case, useZ should be a $(K+1) \times (K+1) \times (N_{\text{mod}} + 1)$ array, where N_{mod} is the number of effect modifiers of A_k. Note that if useZ is specified, its $(k, l, 1)$th entry should equal 1 for all k and l (so that a main effect of A_k is included in the SNCSTM) and the Z component of the data frame called 'data' (see above) should also be specified. See Example 2 for an example of how to specify useZ and Z.

RETURNBOOT : By default, this equals FALSE. To use the *sncstm* function

with the *boot* function, in order to calculate bootstrap confidence intervals, set RETURNBOOT equal to TRUE. See Examples 1 and 2 for how to use the *boot* function.

EXPONCUM : By default, this equals FALSE. If it equals TRUE, the *sncstm* function returns not only the estimates of $\psi_{k(l)}$ $(0 \leqslant k \leqslant l \leqslant K)$ but also the corresponding estimates of $\sum_{j=k}^{l} \psi_{k(j)}$ and $\exp\left\{\sum_{j=k}^{l} \psi_{k(j)}\right\}$. These quantities are relevant to the calculation of relative probabilities of survival (see the analysis of the Cystic Fibrosis data in Section 8 of our article).

VERBOSE : By default, this equals FALSE. If it equals TRUE, the *sncstm* function will print some extra information.

J. Comparison with Picciotto et al.'s (2012) method

J.1 *The case of* $K = 0$

For simplicity, suppose that $K = 0$, that $Z_{0(0)} = 1$ (i.e. no effect modification), that there is no random censoring, and that all individuals still at risk at time 10 are administratively censored at that time.

To compare the SNCSTM with Picciotto et al.'s (2012) method, first reformulate Picciotto et al.'s original structural nested cumulative failure time model as a model for survival, rather than failure. Then treat the data as being the result of ten visits, at times $t = 0, \ldots, 9$, at each of which the exposure of an individual is the same (i.e. A_0), with the exposure effect being $\psi_{0(0)}$. Then Picciotto et al.'s estimating function (see their first equation after their equation (11)) is

$$\sum_{s=0}^{9} R(s)\{A_0 - \hat{E}(A_0 \mid \bar{A}_0^s, L_0, T \geqslant s)\}$$
$$\times \sum_{t=s+1}^{10} J(\bar{A}_0^s, L_0, t) \left\{ \exp\left(\sum_{j=s}^{t-1} \psi_{0(0)} A_0 \right) R(t) - B(\bar{A}_0^s, L_0, t) \right\} \quad (33)$$

where $\bar{A}_0^s = A_0$ if $s > 0$ and is null otherwise, and where $J(\bar{A}_0^s, L_0, t)$ and $B(\bar{A}_0^s, L_0, t)$ are any given functions of $\bar{A}_0^s$, L_0 and t. Picciotto et al. use $J(\bar{A}_0^s, L_0, t) = 1$ and $B(\bar{A}_0^s, L_0, t) = 0$. Obviously, $\hat{E}(A_0 \mid \bar{A}_0^s, L_0, T \geqslant s) = A_0$ when $s > 0$. So, with $B(\bar{A}_0^s, L_0, t) = 0$, expression (33) reduces to

$$\{A_0 - \hat{E}(A_0 \mid L_0)\} \sum_{t=1}^{10} J(L_0, t) \exp(\psi_{0(0)} A_0 t) R(t) \tag{34}$$

As Dukes and Vansteelandt (2018) explain, a drawback of Picciotto et al.'s method relative to Methods 2 and 3 is the difficulty of deriving the efficient choice of $J(L_0, t)$. This difficulty arises because of the correlation between the survival indicators $R(1), \ldots, R(10)$. Methods 2 and 3 are instead based on independent martingale increments, which makes it easier to derive efficient estimating equations.

J.2 *The case of* $K = 1$

Now consider the more complicated scenario where $K = 1$. Suppose that we still have $Z_{0(0)} = Z_{0(1)} = Z_{1(1)} = 1$ (i.e. no effect modification) and still there is no random censoring. Suppose that $S_1 = 10$, i.e. A_1 is measured at time 10 and that all individuals still at risk at time 20 are administratively censored at that time ($S_2 = 20$).

Picciotto et al.'s estimating function for $(\psi_{0(0)}, \psi_{1(1)}, \psi_{0(1)})$ is (using their notation

$H_{st} = H_{st}(\psi_{0(0)}, \psi_{1(1)}, \psi_{0(1)})$ defined on page 890)

$$\sum_{s=0}^{19} R(s) \Big[\{A_0 - \hat{E}(A_0 \mid \bar{A}_0^s, L_0, T \geqslant s)\} I(s \leqslant 9)$$
$$+ \{A_1 - \hat{E}(A_1 \mid \bar{A}_1^s, \bar{L}_1, T \geqslant s)\} I(s \geqslant 10) \Big]$$
$$\times \sum_{t=s+1}^{20} \big\{ J(A_0^s, L_0, t) I(s \leqslant 9) + J(\bar{A}_1^s, \bar{L}_1, t) I(s \geqslant 10) \big\} \times H_{st}$$
$$= \{A_0 - \hat{E}(A_0 \mid L_0)\} \sum_{t=1}^{20} J(L_0, t) H_{0t}$$
$$+ R(10)\{A_1 - \hat{E}(A_1 \mid A_0, \bar{L}_1, T \geqslant 10)\} \sum_{t=11}^{20} J(A_0, \bar{L}_1, t) H_{10,t}$$

where $\bar{A}_1^s = A_0$ for $s \leqslant 10$ and $\bar{A}_1^s = \bar{A}_1$ for $s \geqslant 11$. If we take $J(L_0, t) = J_{0(0)}$ for $t \leqslant 10$, $J(L_0, t) = J_{0(1)}$ for $t \geqslant 11$ and $J(A_0, \bar{L}_1, t) = J_{1(1)}$, then this becomes

$$\{A_0 - \hat{E}(A_0 \mid L_0)\} J_{0(0)} \sum_{t=1}^{10} H_{0t} + \{A_0 - \hat{E}(A_0 \mid L_0)\} J_{0(1)} \sum_{t=11}^{20} H_{0t}$$
$$+ R(10)\{A_1 - \hat{E}(A_1 \mid A_0, \bar{L}_1, T \geqslant 10)\} J_{1(1)} \sum_{t=11}^{20} H_{10,t}$$
$$= \{A_0 - \hat{E}(A_0 \mid L_0)\} J_{0(0)} \sum_{t=1}^{10} \exp(\psi_{0(0)} A_0 t) R(t)$$
$$+ \{A_0 - \hat{E}(A_0 \mid L_0)\} J_{0(1)} \exp\{\psi_{0(0)} A_0 \times 10\} \sum_{t=11}^{20} \exp\{(\psi_{0(1)} A_0 + \psi_{1(1)} A_1)(t - 10)\} R(t)$$
$$+ \{A_1 - \hat{E}(A_1 \mid A_0, \bar{L}_1, T \geqslant 10)\} J_{1(1)} \sum_{t=11}^{20} \exp\{\psi_{1(1)} A_1 (t - 10)\} R(t)$$

If we choose $J_{0(0)} = (1, 0, 0)^\top$, $J_{1(1)} = (0, 1, 0)^\top$ and $J_{0(1)} = (0, 0, 1)^\top$, then this estimating function becomes a vector with the following three elements:

$$\{A_0 - \hat{E}(A_0 \mid L_0)\} \sum_{t=1}^{10} \exp(A_0 \psi_{0(0)} t) R(t)$$
$$\{A_0 - \hat{E}(A_0 \mid L_0)\} \exp\{A_0 \psi_{0(0)} \times 10\} \sum_{t=11}^{20} \exp\{(A_0 \psi_{0(1)} + A_1 \psi_{1(1)})(t - 10)\} R(t)$$
$$\{A_1 - \hat{E}(A_1 \mid A_0, \bar{L}_1, T \geqslant 10)\} \sum_{t=11}^{20} \exp\{A_1 \psi_{1(1)} (t - 10)\} R(t)$$

To impose the constraint that $\psi_{0(0)} = \psi_{1(1)}$, one may instead choose $J_{0(0)} = J_{1(1)} = (1, 0)^\top$ and $J_{0(1)} = (0, 1)^\top$.

J.3 *The general case of $K \geqslant 0$*

More generally, for $K \geqslant 0$, the estimating function is a vector with elements of the form

$$\{A_k - \hat{E}(A_k \mid \bar{A}_{k-1}, \bar{L}_k, T \geqslant 10k)\} \exp\left\{\sum_{j=k}^{l-1} A_j \sum_{m=j}^{l-1} \psi_{j(m)} \times 10\right\}$$
$$\times \sum_{t=10l+1}^{10l+10} \exp\left\{\sum_{j=k}^{l} A_j \psi_{j(l)}(t-10l)\right\} R(t) \qquad (0 \leqslant k \leqslant l \leqslant K)$$

when the constraint $\psi_{k(k+m)} = \psi_{k'(k'+m)}\ \forall k, k', m$ is not imposed, and analogously when the constraint is imposed.

J.4 *Structural nested cumulative failure time model with censoring*

When there is random (i.e. non-administrative) censoring, Picciotto et al. (2012) use inverse probability of censoring weights for their structural nested cumulative failure time model. These weights involve the conditional probability that an individual is censored by time $t+1$ given that he or she has survived and not been censored by time t and his or her treatment and confounder histories at time t. Note that this probability will, in general, depend on the treatment history at time t, even if censoring is completely at random, because individuals cease to be at risk of censoring when they fail. For example, suppose that $K = 0$, that there are no baseline confounders L_0 and that treatment A_0 increases the hazard of failure between times 0 and 1. Then the expected time that an individual is at risk of being censored is less for a treated individual than for an untreated individual. Suppose censoring is completely at random. Then the probability of being censored by time 1 is lower for treated individuals than for untreated individuals, because of the former group's smaller expected time at risk of censoring. For readers who are interested, we now provide a more specific example.

Let A_0 be binary with $P(A_0 = 1) = 0.5$ and let $T(a_0) \sim \exp(0.5+0.5a_0)$ $(a_0 = 0, 1)$

independently of A_0. The true value of $\psi_{0(0)}$ is given by

$$\begin{aligned}\psi_{0(0)} &= \log\left[\frac{P\{T < 1 \mid A_0 = 1\}}{P\{T(0) < 1 \mid A_0 = 1\}}\right] \\ &= \log\left[\frac{P\{T(1) < 1\}}{P\{T(0) < 1\}}\right] \\ &= \log\left[\frac{1 - \exp(-1)}{1 - \exp(-0.5)}\right] \\ &= 0.474.\end{aligned}$$

Picciotto et al.'s estimating function (see their first equation after their equation (11)) is

$$\{A_0 - E(A_0)\}\exp(-\psi_{0(0)}A_0)I(T \leqslant 1) \tag{35}$$

when there is no censoring. Expression (35) can be shown to have expectation zero when $\psi_{0(0)} = 0.474$.

Now suppose there is censoring, with C denoting an individual's censoring time. Picciotto et al. replace the term $I(T \leqslant 1)$ in expression (35) by $I(T \leqslant 1, C > T)$ (see their Section 6) and their estimating function becomes

$$\{A_0 - E(A_0)\}\exp(-\psi_{0(0)}A_0)I(T \leqslant 1, C > T) \tag{36}$$

This equals $-0.5 \times I\{T(0) \leqslant 1, C > T(0)\}$ if $A_0 = 0$ and $0.5\exp(-\psi_{0(0)}) \times I\{T(1) \leqslant 1, C > T(1)\}$ if $A_0 = 1$.

Suppose that $C \sim \exp(5)$ independently of A_0, $T(0)$ and $T(1)$. Then

$$\begin{aligned}E[I\{T(0) \leqslant 1, C > T(0)\}] &= P\{T(0) \leqslant 1, C > T(0)\} \\ &= \int_0^1 \exp\{-(0.5+5)t\} \times 0.5\, dt \\ &= \frac{1 - \exp(-5.5)}{11}\end{aligned}$$

and, similarly,

$$E[I\{T(1) \leqslant 1, C > T(1)\}] = \frac{1 - \exp(-6)}{6}$$

So, the expectation with respect to A_0, T and C of expression (36) is

$$\frac{1}{2}\left[-\frac{1-\exp(-5.5)}{11}\times\frac{1}{2}+\frac{\{1-\exp(-6)\}\exp(-\psi_{0(0)})}{6}\times\frac{1}{2}\right] \tag{37}$$

This equals zero when

$$\psi_{0(0)} = -\log\left\{\frac{6}{11}\times\frac{1-\exp(-5.5)}{1-\exp(-6)}\right\} = 0.608$$

So, the estimator of $\psi_{0(0)}$ will converge asymptotically to 0.608, rather than to the true value, 0.474.

This (asymptotic) bias can be corrected by using inverse probability of censoring weighting. The weight for an individual with $A_0 = a_0$ is $1/P\{C > T(a_0)\} = \exp\{5\,T(a_0)\}$. So, the weighted estimating function is

$$\{A_0 - E(A_0)\}\exp(-\psi_{0(0)}A_0)I(T \leqslant 1, C > T) \times \exp(5T) \tag{38}$$

To calculate the expectation of expression (38) at various values of $\psi_{0(0)}$, we simulated data on A_0, T and C for 10^7 individuals, calculated expression (38) for each individual, and averaged over the individuals. Figure 2 shows the result. Also shown in Figure 2 is the expectation of the unweighted estimating function. (This latter expectation is given by expression (37), but we also verified that the same result was obtained by calculating expression (36) for each of the 10^7 simulated individuals and averaging.) We see from Figure 2 that, unlike the unweighted estimating function, the weighted estimating function has expectation zero at $\psi_{0(0)} = 0.476$. This is very close to the true value of $\psi_{0(0)}$, viz. 0.474. The very small difference is likely to be due to the Monte Carlo error inherent in calculating the expectation by simulation.

J.5 *Censoring for the structural nested model for survival*

Now consider our situation, where Picciotto et al.'s structural nested cumulative failure time model has been reformulated as a model for survival, rather than

for failure. In this reformulated model, no inverse probability of weighting is required when censoring is completely at random. We now explain why this is. Consider the estimating function of expression (34). The quantity $\{A_0 - \hat{E}(A_0 \mid L_0)\}J(L_0, t)\exp(\psi_{0(0)}A_0 t)R(t)$ is unknown if $R(t)$, the survival status at time t, is unknown. If we exclude individuals whose $R(t)$ is unknown, we should compensate for this exclusion by weighting each individual whose $R(t)$ is known by the inverse of the probability that his or her $R(t)$ is known. $R(t)$ is known if $T < C$ or $C > t$. So, if censoring is completely at random, with cumulative hazard $H(t)$, then the probability that $R(t)$ is known is $\exp\{-H(t \wedge T)\}$. In fact, this probability needs to be evaluated only for those individuals whose $R(t)$ is known and equals 1. This is because $\{A_0 - \hat{E}(A_0 \mid L_0)\}J(L_0, t)\exp(\psi_{0(0)}A_0 t)R(t) = 0$ when $R(t) = 0$. For an individual whose $R(t)$ is known to equal 1, T must be greater than t, meaning that $\exp\{-H(t \wedge T)\} = \exp\{-H(t)\}$. Since $\exp\{-H(t)\}$ is a constant (apart from depending on t), there is no need to weight by its inverse.

J.6 *Applying Picciotto et al.'s estimation method in simulation study*

We compared the performance of Picciotto et al.'s estimation method to our Methods 1–3 in the two regular visit scenarios of the simulation study of Section 7 of our article. In this simulation study, $S_1 = 1$, $S_2 = 2$, etc., rather than $S_1 = 10$, $S = 20$, etc. as was assumed above. So, the estimating equations become

$$\{A_0 - \hat{E}(A_0 \mid L_0)\} \sum_{t=1}^{10} \exp(A_0\psi_{0(0)}t/10)R(t/10) = 0$$

$$\{A_0 - \hat{E}(A_0 \mid L_0)\} \exp\{A_0\psi_{0(0)}\} \sum_{t=11}^{20} \exp\{(A_0\psi_{0(1)} + A_1\psi_{1(1)})(t-10)/10\}R(t/10) = 0$$

$$\{A_1 - \hat{E}(A_1 \mid A_0, \bar{L}_1, T \geqslant 1)\} \sum_{t=11}^{20} \exp\{A_1\psi_{1(1)}(t-10)/10\}R(t/10) = 0$$

etc., when the constraint $\psi_{k(k+m)} = \psi_{k'(k'+m)}\ \forall k, k', m$ is not imposed, and analogously when the constraint is imposed. Inverse probability of censoring weighting was used in the scenario with random censoring, as described in Web Appendix J.4.

As Picciotto et al. (2012) note, these estimating equations cannot be solved using the Newton-Raphson algorithm. They used the Newson-Mead algorithm; we used a simple grid search with a fine grid.

Results are reported in Section 7 and Tables 1 and 2 of our article.

K. Competing risks

Suppose there are two competing causes of failure. Let $T(\bar{A}_k, 0)$ be the counterfactual failure time as previously defined, that is, it is the time from the start of the study until failure, regardless of the cause of failure. Let $N^{(j)}_{(\bar{A}_k,0)}(t)$ denote the counterfactual counting process indicator for a failure due to cause j ($j = 1, 2$). We consider the following semi-parametric additive cause-specific hazard model

$$
\begin{aligned}
&E\left\{dN^{(j)}_{(\bar{A}_{k-1},0)}(t) \mid \bar{A}_k, \bar{L}_k, T(\bar{A}_{k-1}, 0) \geqslant t\right\} \\
&= E\left\{dN^{(j)}_{(\bar{A}_k,0)}(t) \mid \bar{A}_k, \bar{L}_k, T(\bar{A}_k, 0) \geqslant t\right\} - A_k \psi^{(j)}_{k(l)} dt,
\end{aligned}
$$

for $t \in [l, l+1)$ and $j = 1, 2$. Building on similar results in Martinussen and Vansteelandt (2018), the procedure proposed in our article is readily adjusted to the estimation of $\psi^{(j)}_{k(l)}$ ($j = 1, 2$) by redefining $w_k(t)$ as

$$
w_k(t) = \prod_{j=k+1}^{K} \exp\left\{A_k \psi^{(1)}_{k(l)} + A_k \psi^{(2)}_{k(l)}\right\}.
$$

This change accounts for the fact that the conditional mean of the at-risk indicator $R(t) = I(T \geqslant t)$ is influenced by the cause-specific hazards of both failure types. For instance, equation (7) becomes

$$
\sum_{i=1}^{n} \int_{l}^{l+1} R_i(t) w_{ki}(t) \Delta_{ki}(t) \left[dN^{(j)}_i(t) - \left\{ \sum_{s=k+1}^{l} A_{si} \psi^{(j)}_{s(l)} + \Delta_{ki}(l) \psi^{(j)}_{k(l)} \right\} dt \right] = 0,
$$

which must be solved jointly for $j = 1$ and $j = 2$.

Formulae very similar to those given by Martinussen and Vansteelandt (2018) can be used to convert these estimates of the parameters $\psi_{k(l)}^{(j)}$, which describe the causal effect of exposure on cause-specific hazards, into estimates of the causal effect of exposure on cumulative incidence.

L. Additional simulation studies

In Section 7 we showed results for $n = 1000$ in the two regular visit scenarios, one with no censoring and one with random censoring. Web Tables 4 and 5 show the results for the irregular visit scenario.

Web Tables 6–11 show the corresponding results for the three scenarios when $n = 250$.

Web Tables 12–17 show the corresponding results for $n = 1000$ with a shorter follow-up time. In this case, the visit times are divided by four and administrative censoring occurs at time 1. This means that the visit times are $S_k = k/4$ ($k = 0, \ldots, 4$) in the regular visit scenarios and the inter-visit times are $S_k - S_{k-1} \sim \text{Uniform}[0.5/4, 1.5/4]$ in the irregular visit scenario.

We also carried out a simple simulation study to illustrate the double robustness properties of Methods 2 and 3. We shall use $\mathcal{B}^*_{k(l)}$ to denote the modified version of Model $\mathcal{B}_{k(l)}$ allowed by Method 3. The original model, Model $\mathcal{B}_{k(l)}$, allowed by Method 2 assumes that the intercept and coefficients for $\bar{A}_{k-1}$ and $\bar{L}_k$ are constant over time. The modified model, Model $\mathcal{B}^*_{k(l)}$, is more general, allowing as it does the intercept and coefficients for $\bar{A}_{k-1}$ and $\bar{L}_k$ to vary over time. If Model $\mathcal{B}_{k(l)}$ is correctly specified, then so is Model $\mathcal{B}^*_{k(l)}$.

In this simulation study, we assumed $K = 1$, $S_0 = 0$, $S_1 = 1$, a single time-

dependent, continuous confounder, and a continuous treatment. The hazard of $T(a_0, 0)$ given A_0 and L_0 during time interval $t \in [0, 1)$ was assumed to be

$$E\{dN_{(a_0,0)}(t) \mid A_0, L_0, T(a_0, 0) \geqslant t\} = \omega_0 + \gamma_{00}L_0 + \delta_{00}a_0 \qquad t \in [0, 1)$$

where $\omega_0 = 2.7$, $\gamma_{00} = 0.75$ and $\delta_{00} = -0.3$. This implies that Model $\mathcal{M}_0$ is correctly specified during the time interval $t \in [0, 1)$ and $\psi_{0(0)} = \delta_{00} = -0.3$. It also implies that Model $\mathcal{B}_{0(0)}$ is correctly specified, since $E\{dN_{(0)}(t) \mid A_0, L_0, T(0) \geqslant t\} = \omega_0 + \gamma_{00}L_0$ is linear in L_0 and the intercept ω_0 and coefficient γ_{00} of L_0 are constant in t.

Intervening on A_0 may change the value of L_1. So, let $L_1(a_0)$ denote the value of L_1 when A_0 is set by intervention to equal a_0. We shall assume that

$$L_1(a_0) \mid A_0 = a_0, L_0, T(a_0, 0) \geqslant 1 \sim N(1.6 - 0.5a_0, 0.5)$$

The hazard of $T(a_0, a_1)$ given $\bar{A}_1$, L_0 and $L_1(a_0)$ during time interval $t \in [1, 2)$ was assumed to be

$$\begin{aligned} &E\{dN_{(a_0,a_1)}(t) \mid \bar{A}_1 = (a_0, a_1), L_0, L(a_0), T(a_0, a_1) \geqslant t\} \\ &= \omega_1 + \gamma_{10}L_0 + \gamma_{11}L_1(a_0) + \delta_{10}a_0 + \delta_{11}a_1 + (t-1)c \qquad t \in [1, 2) \end{aligned}$$

where $\omega_1 = 2.7$, $\gamma_{10} = 0$, $\gamma_{11} = 0.75$, $\delta_{10} = 0.275$, $\delta_{11} = -0.3$ and c is a constant. We shall consider two values of c: 0 and $9/32$. This form of the hazard implies that Model $\mathcal{M}_1$ is correctly specified and $\psi_{1(1)} = \delta_{11} = -0.3$. Also, Model $\mathcal{B}_{0(0)}$ is correctly specified if $c = 0$, but not if $c = 9/32$ (because the intercept term $2.7 + (t-1)c$ is then a function of t). However, Model $\mathcal{B}^*_{0(0)}$ is correctly specified regardless of whether $c = 0$ or $c = 9/32$ (because the intercept in that model is allowed to be a function of t).

We shall now show that: i) Model $\mathcal{M}_0$ is correctly specified during time interval

$t \in [1,2)$, with $\psi_{0(1)} = -0.1$; ii) Model $\mathcal{B}_{0(1)}$ is correctly specified if $c = 9/32$ but not if $c = 0$; and iii) Model $\mathcal{B}^*_{0(1)}$ is correctly specified whatever the value of c.

For $t \in [1,2)$,

$$\begin{aligned}
&P\{T(a_0,0) \geqslant t \mid A_0, L_0\} \\
&= P\{T(a_0,0) \geqslant 1 \mid A_0, L_0\} \\
&\quad \times E_{L_1(a_0)}\left[P\{T(a_0,0) \geqslant t \mid \bar{A}_1, L_1(a_0), T(a_0,0) \geqslant 1\} \mid A_0, L_0, T(a_0,0) \geqslant 1\right] \\
&= \exp\{-(\omega_0 + \gamma_{00}L_0 + \delta_{00}a_0)\} \\
&\quad \times E_{L_1(a_0)}\left(\exp\left[-\{\omega_1 + (t-1)c/2 + \gamma_{10}L_0 + \gamma_{11}L_1(a_0) + \delta_{10}a_0\}(t-1)\right]\right. \\
&\qquad\qquad\qquad\qquad\qquad\qquad\qquad\qquad\left. \mid A_0, L_0, T(a_0,0) \geqslant 1\right) \\
&= \exp\left[-\{\omega_0 + \gamma_{00}L_0 + \delta_{00}a_0\} - \{\omega_1 + (t-1)c/2 + \gamma_{10}L_0 + \delta_{10}a_0\}(t-1)\right] \\
&\quad \times E_{L_1(a_0)}\left[\exp\{-\gamma_{11}L_1(a_0)\,(t-1)\} \mid A_0, L_0, T(a_0,0) \geqslant 1\right]
\end{aligned} \tag{39}$$

Moreover, because $L_1(a_0)$ is normally distributed given A_0, L_0 and $T(a_0,0) \geqslant 1$, and using the form of the moment generating function of a normal distribution, we have

$$\begin{aligned}
&E_{L_1(a_0)}\left[\exp\{-\gamma_{11}L_1(a_0)\,(t-1)\} \mid A_0, L_0, T(a_0,0) \geqslant 1\right] \\
&= \exp\left[-E\{\gamma_{11}L_1(a_0)(t-1) \mid A_0, L_0, T(a_0,0) \geqslant 1\}\right. \\
&\qquad\left. + \frac{1}{2}\mathrm{Var}\{\gamma_{11}L_1(a_0)(t-1) \mid A_0, L_0, T(a_0,0) \geqslant 1\}\right] \\
&= \exp\left[-(t-1)\gamma_{11}E\{L_1(a_0) \mid A_0, L_0, T(a_0,0) \geqslant 1\}\right. \\
&\qquad\left. + \frac{1}{2}(t-1)^2\gamma_{11}^2\mathrm{Var}\{L_1(a_0) \mid A_0, L_0, T(a_0,0) \geqslant 1\}\right]
\end{aligned} \tag{40}$$

It follows from equations (39) and (40) that the conditional hazard of $T(a_0,0)$ given A_0 and L_0 during time interval $t \in [1,2)$ equals

$$\begin{aligned}
&\omega_1 + (t-1)c + \gamma_{10}L_0 + \delta_{10}a_0 + \gamma_{11}E\{L_1(a_0) \mid A_0, L_0, T(a_0,0) \geqslant 1\} \\
&-(t-1)\gamma_{11}^2\mathrm{Var}\{L_1(a_0) \mid A_0, L_0, T(a_0,0) \geqslant 1\}
\end{aligned} \tag{41}$$

We see from the hazard (41) that Model $\mathcal{M}_0$ is correctly specified in the time interval $t \in [1, 2)$, with

$$\begin{aligned}\psi_{0(1)} &= \delta_{10} + \gamma_{11} \times \frac{E\{L_1(a_0) \mid A_0, L_0\} - E\{L_1(0) \mid A_0, L_0\}}{a_0} \\ &= 0.275 + 0.75 \times (-0.5) \\ &= -0.1\end{aligned}$$

Moreover, if $c = \gamma_{11}^2 \mathrm{Var}\{L_1(a_0) \mid A_0, L_0, T(a_0, 0) \geqslant 1\} = 0.75^2 \times 0.5 = 9/32$, the hazard (41) reduces to

$$\begin{aligned}&\omega_1 + \gamma_{10}L_0 + \delta_{10}a_0 + \gamma_{11}E\{L_1(a_0) \mid A_0, L_0, T(a_0, 0) \geqslant 1\} \\ &= 2.7 + 0 \times L_0 + 0.275a_0 + 0.75(1.6 - 0.5a_0) \\ &= 3.9 - 0.1a_0\end{aligned}$$

which shows that Model $\mathcal{B}_{0(1)}$ is correctly specified if $c = 9/32$ but not if $c = 0$. The more general model, Model $\mathcal{B}^*_{0(1)}$, is correctly specified whether $c = 0$ or $c = 9/32$.

We generated observed data from this model as follows. First, we need to specify how to generate L_0, A_0 and A_1.

For L_0, we assumed that $L_0 \sim N(0, 0.5)$.

The data-generating distribution of A_0 given L_0 was either $A_0 \mid L_0 \sim N(3 - L_0, 0.9^2)$ or $A_0 \mid L_0 \sim N(3.5 - 2I(L_0 \geqslant 0.75), 0.9^2)$, where $I(.)$ is the indicator function. We call these two data-generating models $\mathcal{A}_0^{\mathrm{gen}(1)}$ and $\mathcal{A}_0^{\mathrm{gen}(2)}$, respectively. The Model $\mathcal{A}_0$ that will be assumed when fitting the SNCSTM to these simulated data is a linear regression of A_0 on L_0. So, if the data-generating model is $\mathcal{A}_0^{\mathrm{gen}(1)}$ then the assumed Model $\mathcal{A}_0$ is correctly specified, but if it is $\mathcal{A}_0^{\mathrm{gen}(2)}$ then Model $\mathcal{A}_0$ is misspecified.

The hazard for the observed failure time T is $2.7 + 0.75L_0 - 0.3A_0$ during the time interval $t \in [0, 1)$.

To generate L_1 given A_0, L_1 and $T \geqslant 1$, we assumed

$$L_1 \mid A_0, L_0, T \geqslant 1 \sim N(1.6 - 0.5A_0, 0.5)$$

The true data-generating distribution of A_1 given A_0, $\bar{L}_1$ and $T \geqslant 1$ was either $A_1 \mid A_0, \bar{L}_1, T \geqslant 1 \sim N(3 - L_1, 0.9^2)$ (called model $\mathcal{A}_1^{\text{gen}(1)}$) or $A_1 \mid A_0, \bar{L}_1, T \geqslant 1 \sim N(3.5 - 2I(L_1 \geqslant 0.75), 0.9^2)$ (called model $\mathcal{A}_1^{\text{gen}(2)}$). The Model $\mathcal{A}_1$ that will be assumed when fitting the SNCSTM to these simulated data is a linear regression of A_1 on A_0 and $\bar{L}_1$. So, if the data-generating model is $\mathcal{A}_1^{\text{gen}(1)}$ then Model $\mathcal{A}_1$ is correctly specified, but if it is $\mathcal{A}_1^{\text{gen}(2)}$ then Model $\mathcal{A}_1$ is misspecified.

The hazard for the observed failure time T is $2.7+0.75L_1+0.275A_0-0.3A_1+(t-1)c$ during the time interval $t \in [1, 2)$.

We considered the following four scenarios:

(1) The data-generating models for A_0 and A_1 are $\mathcal{A}_0^{\text{gen}(1)}$ and $\mathcal{A}_1^{\text{gen}(1)}$. This means that Models $\mathcal{A}_0$ and $\mathcal{A}_1$ are correctly specified, and so all of Methods 1–3 should yield consistent estimates of $\psi_{0(0)}$, $\psi_{0(1)}$ and $\psi_{1(1)}$. Here we used $c = 0$.

(2) The data-generating models for A_0 and A_1 are $\mathcal{A}_0^{\text{gen}(1)}$ and $\mathcal{A}_1^{\text{gen}(2)}$. This means that $\mathcal{A}_0$ is correctly specified, but $\mathcal{A}_1$ is misspecified. Here, Method 1 should yield a consistent estimate of $\psi_{0(0)}$ but the estimates of $\psi_{0(1)}$ and $\psi_{1(1)}$ may be inconsistent. We used $c = 0$, which means Model $\mathcal{B}_{1(1)}$ is also correctly specified. The double robustness properties of Methods 2 and 3 should mean that these methods yield consistent estimates not only of $\psi_{0(0)}$ but also of $\psi_{0(1)}$ and $\psi_{1(1)}$.

(3) The data-generating models for A_0 and A_1 are $\mathcal{A}_0^{\text{gen}(2)}$ and $\mathcal{A}_1^{\text{gen}(1)}$. This means that $\mathcal{A}_0$ is misspecified, but $\mathcal{A}_1$ is correctly specified. Here, Method 1 should yield a consistent estimate of $\psi_{1(1)}$ but the estimates of $\psi_{0(0)}$ and $\psi_{0(1)}$ may be inconsistent. We used $c = 9/32$, which means Model $\mathcal{B}_{0(1)}$ is correctly specified.

Since Model $\mathcal{B}_{0(0)}$ is also correctly specified, the double robustness properties of Methods 2 and 3 should mean that these methods yield consistent estimates of $\psi_{0(0)}$, $\psi_{0(1)}$ and $\psi_{1(1)}$.

(4) The data-generating models for A_0 and A_1 are $\mathcal{A}_0^{\text{gen}(2)}$ and $\mathcal{A}_1^{\text{gen}(2)}$. This means that Models $\mathcal{A}_0$ and $\mathcal{A}_1$ are both misspecified. Here, Method 1 may yield inconsistent estimates of all three parameters. We used $c = 0$, which means that Models $\mathcal{B}_{1(1)}$ and $\mathcal{B}_{0(0)}$ are correctly specified. Method 2 should therefore yield consistent estimates of $\psi_{0(0)}$ and $\psi_{1(1)}$. However, Model $\mathcal{B}_{0(1)}$ is misspecified, meaning that the estimate of $\psi_{0(1)}$ from Method 2 may be inconsistent. Method 3 should yield consistent estimates of all three parameters, because Model $\mathcal{B}^*_{0(1)}$ is correctly specified.

For each of these four scenarios, we considered the case of no random censoring and the case of censoring completely at random with constant censoring hazard 0.5. Thus, there were a total of eight scenarios. In all eight scenarios, all individuals who had not failed or been censored prior to time $t = 2$ were administratively censored at that time. For each scenario, we generated 5000 simulated datasets, each of $n = 5000$ individuals.

Table 18 shows the mean of the parameter estimates over the 5000 simulated datasts when there is no censoring. Results for the random censoring scenarios were very similar. Monte Carlo standard errors for these means are shown in brackets. The results are as expected. That is, where we have predicted an estimator to be consistent, it is approximately unbiased, and where it has been predicted to be possibly inconsistent, it is generally biased. One exception is that the bias (if any) in the estimator of $\psi_{0(1)}$ from Method 2 is very small even in the fourth scenario, where we predicted this estimator was likely to be inconsistent. This is probably

because the misspecification of Model $\mathcal{B}_{0(1)}$ is only minor, i.e. only the intercept term depends on t and this dependence is not large.

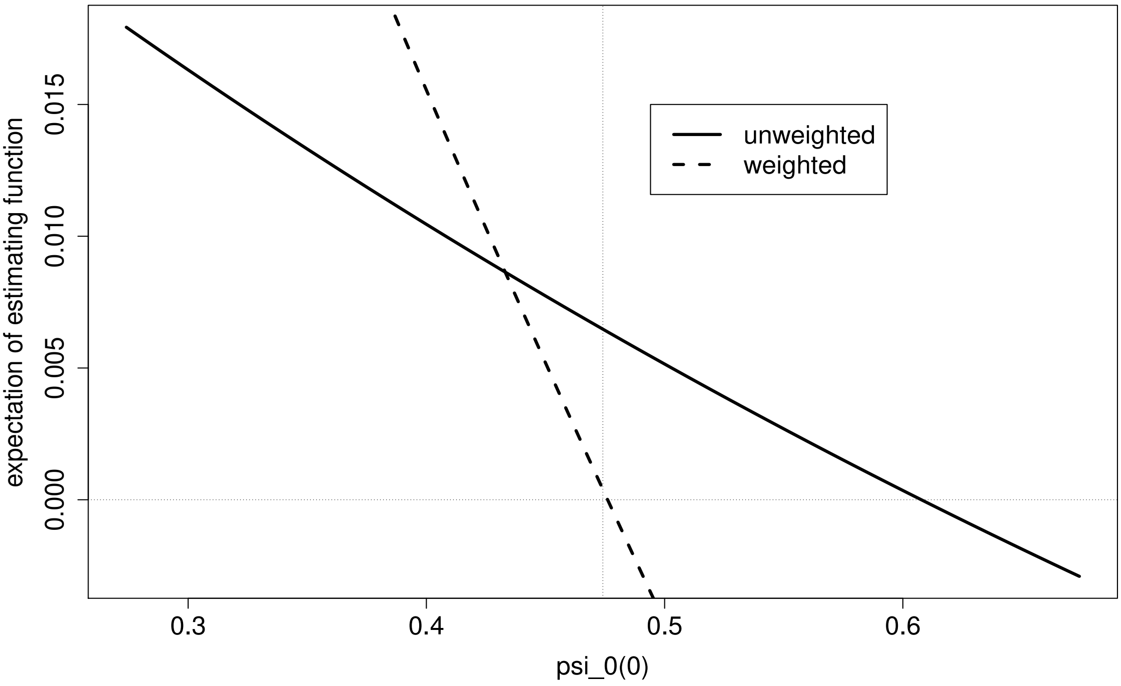

Figure 2. Expectations of unweighted (solid line) and weighted estimating function (broken line) as functions of $\psi_{0(0)}$. The true value of $\psi_{0(0)}$ is shown by the vertical dotted line.

Mtd	Con	$\psi_{0(0)}$	$\psi_{0(1)}$	$\psi_{0(2)}$	$\psi_{0(3)}$	$\psi_{1(1)}$	$\psi_{1(2)}$	$\psi_{1(3)}$	$\psi_{2(2)}$	$\psi_{2(3)}$	$\psi_{3(3)}$
True		0.400	0.100	0.040	0.020	0.400	0.100	0.040	0.400	0.100	0.400
1	no	0.394	0.112	0.009	0.024	0.399	0.118	0.034	0.382	0.133	0.354
1cw	no	0.390	0.110	0.009	0.025	0.418	0.106	0.036	0.397	0.136	0.351
2	no	0.398	0.110	0.034	0.026	0.401	0.096	0.020	0.413	0.114	0.408
1	yes	0.392	0.116	0.026	0.024	0.392	0.116	0.026	0.392	0.116	0.392
1cw	yes	0.395	0.112	0.027	0.025	0.395	0.112	0.027	0.395	0.112	0.395
2	yes	0.401	0.107	0.029	0.027	0.401	0.107	0.029	0.401	0.107	0.401

Table 4

Means (×10) of parameter estimates when n=1000 and visits are irregular and there is random censoring. 'Mtd' is method ('1cw' is Method 1 with censoring weighting) and 'Con' is whether constraint $\psi_{k(k+m)} = \psi_{k'(k'+m)}$ is imposed.

Mtd	Con	$\psi_{0(0)}$	$\psi_{0(1)}$	$\psi_{0(2)}$	$\psi_{0(3)}$	$\psi_{1(1)}$	$\psi_{1(2)}$	$\psi_{1(3)}$	$\psi_{2(2)}$	$\psi_{2(3)}$	$\psi_{3(3)}$
1	no	0.486	0.645	0.828	0.555	0.722	1.044	1.013	0.875	1.144	1.057
1cw	no	0.453	0.617	0.831	0.556	0.656	1.001	1.015	0.797	1.098	0.997
2	no	0.180	0.214	0.245	0.277	0.265	0.315	0.378	0.305	0.374	0.355
1	yes	0.355	0.490	0.646	0.543	0.355	0.490	0.646	0.355	0.490	0.355
1cw	yes	0.325	0.465	0.646	0.545	0.325	0.465	0.646	0.325	0.465	0.325
2	yes	0.125	0.161	0.204	0.273	0.125	0.161	0.204	0.125	0.161	0.125

Table 5

SEs ($\times 10$) of parameter estimates when n=1000 and visits are irregular and there is random censoring. 'Mtd' is method ('1cw' is Method 1 with censoring weighting) and 'Con' is whether constraint $\psi_{k(k+m)} = \psi_{k'(k'+m)}$ is imposed.

Mtd	Con	$\psi_{0(0)}$	$\psi_{0(1)}$	$\psi_{0(2)}$	$\psi_{0(3)}$	$\psi_{1(1)}$	$\psi_{1(2)}$	$\psi_{1(3)}$	$\psi_{2(2)}$	$\psi_{2(3)}$	$\psi_{3(3)}$
True		0.400	0.100	0.040	0.020	0.400	0.100	0.040	0.400	0.100	0.400
1	no	0.396	0.098	0.034	0.037	0.397	0.118	0.005	0.393	0.100	0.375
2	no	0.405	0.100	0.042	0.037	0.423	0.128	0.013	0.423	0.106	0.408
3	no	0.403	0.099	0.042	0.037	0.417	0.125	0.014	0.412	0.103	0.393
1	yes	0.362	0.099	0.028	0.037	0.362	0.099	0.028	0.362	0.099	0.362
2	yes	0.410	0.109	0.037	0.036	0.410	0.109	0.037	0.410	0.109	0.410
3	yes	0.403	0.107	0.037	0.036	0.403	0.107	0.037	0.403	0.107	0.403

Table 6

Means (×10) of parameter estimates when n=250 and visits are regular and there is no random censoring. 'Mtd' is method ('1cw' is Method 1 with censoring weighting) and 'Con' is whether constraint $\psi_{k(k+m)} = \psi_{k'(k'+m)}$ *is imposed.*

Mtd	Con	$\psi_{0(0)}$	$\psi_{0(1)}$	$\psi_{0(2)}$	$\psi_{0(3)}$	$\psi_{1(1)}$	$\psi_{1(2)}$	$\psi_{1(3)}$	$\psi_{2(2)}$	$\psi_{2(3)}$	$\psi_{3(3)}$
1	no	0.377	0.372	0.416	0.448	0.473	0.492	0.543	0.514	0.571	0.549
2	no	0.363	0.359	0.407	0.439	0.474	0.491	0.540	0.521	0.584	0.574
3	no	0.361	0.356	0.404	0.433	0.467	0.482	0.528	0.508	0.568	0.554
1	yes	0.227	0.249	0.304	0.443	0.227	0.249	0.304	0.227	0.249	0.227
2	yes	0.235	0.255	0.310	0.434	0.235	0.255	0.310	0.235	0.255	0.235
3	yes	0.232	0.251	0.306	0.429	0.232	0.251	0.306	0.232	0.251	0.232

Table 7

SEs ($\times 10$) of parameter estimates when n=250 and visits are regular and there is no random censoring. 'Mtd' is method ('1cw' is Method 1 with censoring weighting) and 'Con' is whether constraint $\psi_{k(k+m)} = \psi_{k'(k'+m)}$ is imposed.

Mtd	Con	$\psi_{0(0)}$	$\psi_{0(1)}$	$\psi_{0(2)}$	$\psi_{0(3)}$	$\psi_{1(1)}$	$\psi_{1(2)}$	$\psi_{1(3)}$	$\psi_{2(2)}$	$\psi_{2(3)}$	$\psi_{3(3)}$
True		0.400	0.100	0.040	0.020	0.400	0.100	0.040	0.400	0.100	0.400
1	no	0.409	0.115	0.015	-0.003	0.388	0.076	-0.034	0.397	0.153	0.354
1cw	no	0.399	0.107	0.021	-0.005	0.373	0.105	-0.037	0.408	0.121	0.354
2	no	0.404	0.107	0.050	0.051	0.419	0.115	0.020	0.439	0.101	0.403
3	no	0.401	0.106	0.050	0.049	0.411	0.114	0.024	0.422	0.094	0.376
1	yes	0.382	0.108	0.005	-0.002	0.382	0.108	0.005	0.382	0.108	0.382
1cw	yes	0.373	0.105	0.010	-0.005	0.373	0.105	0.010	0.373	0.105	0.373
2	yes	0.409	0.108	0.049	0.046	0.409	0.108	0.049	0.409	0.108	0.409
3	yes	0.401	0.106	0.049	0.044	0.401	0.106	0.049	0.401	0.106	0.401

Table 8

Means (×10) of parameter estimates when n=250 and visits are regular and there is random censoring. 'Mtd' is method ('1cw' is Method 1 with censoring weighting) and 'Con' is whether constraint $\psi_{k(k+m)} = \psi_{k'(k'+m)}$ *is imposed.*

Mtd	Con	$\psi_{0(0)}$	$\psi_{0(1)}$	$\psi_{0(2)}$	$\psi_{0(3)}$	$\psi_{1(1)}$	$\psi_{1(2)}$	$\psi_{1(3)}$	$\psi_{2(2)}$	$\psi_{2(3)}$	$\psi_{3(3)}$
1	no	0.544	0.638	0.765	0.948	0.840	0.949	1.197	0.967	1.161	1.203
1cw	no	0.431	0.482	0.778	0.974	0.633	0.711	1.230	0.741	0.926	0.911
2	no	0.387	0.422	0.524	0.671	0.585	0.659	0.866	0.707	0.885	0.898
3	no	0.385	0.418	0.516	0.655	0.575	0.641	0.832	0.683	0.830	0.840
1	yes	0.383	0.474	0.616	0.902	0.383	0.474	0.616	0.383	0.474	0.383
1cw	yes	0.294	0.355	0.632	0.938	0.294	0.355	0.632	0.294	0.355	0.294
2	yes	0.280	0.325	0.432	0.644	0.280	0.325	0.432	0.280	0.325	0.280
3	yes	0.276	0.317	0.424	0.630	0.276	0.317	0.424	0.276	0.317	0.276

Table 9

SEs (×10) of parameter estimates when n=250 and visits are regular and there is random censoring. 'Mtd' is method ('1cw' is Method 1 with censoring weighting) and 'Con' is whether constraint $\psi_{k(k+m)} = \psi_{k'(k'+m)}$ is imposed.

Mtd	Con	$\psi_{0(0)}$	$\psi_{0(1)}$	$\psi_{0(2)}$	$\psi_{0(3)}$	$\psi_{1(1)}$	$\psi_{1(2)}$	$\psi_{1(3)}$	$\psi_{2(2)}$	$\psi_{2(3)}$	$\psi_{3(3)}$
True		0.400	0.100	0.040	0.020	0.400	0.100	0.040	0.400	0.100	0.400
1	no	0.448	0.074	0.043	-0.045	0.387	0.161	0.028	0.294	0.126	0.374
1cw	no	0.440	0.089	0.044	-0.049	0.381	0.146	0.025	0.289	0.124	0.400
2	no	0.423	0.090	0.043	-0.022	0.439	0.124	0.054	0.422	0.113	0.442
1	yes	0.403	0.117	0.030	-0.029	0.403	0.117	0.030	0.403	0.117	0.403
1cw	yes	0.401	0.120	0.030	-0.031	0.401	0.120	0.030	0.401	0.120	0.401
2	yes	0.425	0.099	0.045	-0.021	0.425	0.099	0.045	0.425	0.099	0.425

Table 10

Means (×10) of parameter estimates when n=250 and visits are irregular and there is random censoring. 'Mtd' is method ('1cw' is Method 1 with censoring weighting) and 'Con' is whether constraint $\psi_{k(k+m)} = \psi_{k'(k'+m)}$ is imposed.

Mtd	Con	$\psi_{0(0)}$	$\psi_{0(1)}$	$\psi_{0(2)}$	$\psi_{0(3)}$	$\psi_{1(1)}$	$\psi_{1(2)}$	$\psi_{1(3)}$	$\psi_{2(2)}$	$\psi_{2(3)}$	$\psi_{3(3)}$
1	no	0.956	1.356	1.661	1.253	1.422	2.148	1.941	1.746	2.309	2.001
1cw	no	0.885	1.315	1.670	1.266	1.344	2.059	1.972	1.639	2.238	1.895
2	no	0.381	0.433	0.533	0.715	0.558	0.715	0.898	0.732	0.943	0.892
1	yes	0.695	1.039	1.234	1.135	0.695	1.039	1.234	0.695	1.039	0.695
1cw	yes	0.646	1.000	1.256	1.161	0.646	1.000	1.256	0.646	1.000	0.646
2	yes	0.262	0.337	0.450	0.687	0.262	0.337	0.450	0.262	0.337	0.262

Table 11

SEs (×10) of parameter estimates when n=250 and visits are irregular and there is random censoring. 'Mtd' is method ('1cw' is Method 1 with censoring weighting) and 'Con' is whether constraint $\psi_{k(k+m)} = \psi_{k'(k'+m)}$ is imposed.

Mtd	Con	$\psi_{0(0)}$	$\psi_{0(1)}$	$\psi_{0(2)}$	$\psi_{0(3)}$	$\psi_{1(1)}$	$\psi_{1(2)}$	$\psi_{1(3)}$	$\psi_{2(2)}$	$\psi_{2(3)}$	$\psi_{3(3)}$
True		0.400	0.100	0.040	0.020	0.400	0.100	0.040	0.400	0.100	0.400
1	no	0.393	0.099	0.028	0.005	0.391	0.070	0.036	0.396	0.111	0.386
2	no	0.396	0.103	0.026	0.009	0.395	0.071	0.035	0.406	0.109	0.393
3	no	0.396	0.103	0.026	0.009	0.394	0.071	0.035	0.404	0.109	0.390
1	yes	0.369	0.089	0.030	0.004	0.369	0.089	0.030	0.369	0.089	0.369
2	yes	0.397	0.096	0.029	0.008	0.397	0.096	0.029	0.397	0.096	0.397
3	yes	0.396	0.096	0.029	0.008	0.396	0.096	0.029	0.396	0.096	0.396

Table 12

Means ($\times 10$) of parameter estimates when n=1000, times between visits are divided by four and visits are regular and there is no random censoring. 'Mtd' is method ('1cw' is Method 1 with censoring weighting) and 'Con' is whether constraint $\psi_{k(k+m)} = \psi_{k'(k'+m)}$ is imposed.

Mtd	Con	$\psi_{0(0)}$	$\psi_{0(1)}$	$\psi_{0(2)}$	$\psi_{0(3)}$	$\psi_{1(1)}$	$\psi_{1(2)}$	$\psi_{1(3)}$	$\psi_{2(2)}$	$\psi_{2(3)}$	$\psi_{3(3)}$
1	no	0.355	0.339	0.318	0.326	0.433	0.428	0.428	0.431	0.427	0.422
2	no	0.342	0.323	0.310	0.312	0.417	0.407	0.415	0.409	0.407	0.411
3	no	0.341	0.322	0.310	0.312	0.416	0.405	0.413	0.407	0.405	0.409
1	yes	0.201	0.218	0.245	0.325	0.201	0.218	0.245	0.201	0.218	0.201
2	yes	0.200	0.216	0.243	0.311	0.200	0.216	0.243	0.200	0.216	0.200
3	yes	0.200	0.215	0.242	0.311	0.200	0.215	0.242	0.200	0.215	0.200

Table 13

SEs (×10) of parameter estimates when n=1000, times between visits are divided by four and visits are regular and there is no random censoring. 'Mtd' is method ('1cw' is Method 1 with censoring weighting) and 'Con' is whether constraint $\psi_{k(k+m)} = \psi_{k'(k'+m)}$ *is imposed.*

Mtd	Con	$\psi_{0(0)}$	$\psi_{0(1)}$	$\psi_{0(2)}$	$\psi_{0(3)}$	$\psi_{1(1)}$	$\psi_{1(2)}$	$\psi_{1(3)}$	$\psi_{2(2)}$	$\psi_{2(3)}$	$\psi_{3(3)}$
True		0.400	0.100	0.040	0.020	0.400	0.100	0.040	0.400	0.100	0.400
1	no	0.391	0.093	0.027	0.021	0.394	0.068	0.028	0.399	0.088	0.396
1cw	no	0.396	0.100	0.027	0.020	0.398	0.063	0.028	0.408	0.099	0.400
2	no	0.394	0.104	0.028	0.009	0.398	0.073	0.044	0.404	0.104	0.401
3	no	0.394	0.104	0.027	0.009	0.396	0.073	0.044	0.402	0.104	0.398
1	yes	0.381	0.082	0.026	0.020	0.381	0.082	0.026	0.381	0.082	0.381
1cw	yes	0.387	0.087	0.027	0.019	0.387	0.087	0.027	0.387	0.087	0.387
2	yes	0.398	0.096	0.034	0.008	0.398	0.096	0.034	0.398	0.096	0.398
3	yes	0.396	0.096	0.034	0.008	0.396	0.096	0.034	0.396	0.096	0.396

Table 14

Means (×10) of parameter estimates when n=1000, times between visits are divided by four and visits are regular and there is random censoring. 'Mtd' is method ('1cw' is Method 1 with censoring weighting) and 'Con' is whether constraint $\psi_{k(k+m)} = \psi_{k'(k'+m)}$ is imposed.

Mtd	Con	$\psi_{0(0)}$	$\psi_{0(1)}$	$\psi_{0(2)}$	$\psi_{0(3)}$	$\psi_{1(1)}$	$\psi_{1(2)}$	$\psi_{1(3)}$	$\psi_{2(2)}$	$\psi_{2(3)}$	$\psi_{3(3)}$
1	no	0.502	0.507	0.502	0.532	0.635	0.672	0.693	0.669	0.677	0.680
1cw	no	0.380	0.378	0.503	0.534	0.471	0.487	0.694	0.481	0.508	0.485
2	no	0.346	0.332	0.330	0.339	0.429	0.433	0.449	0.430	0.452	0.444
3	no	0.346	0.332	0.329	0.338	0.427	0.431	0.447	0.428	0.450	0.440
1	yes	0.302	0.339	0.402	0.530	0.302	0.339	0.402	0.302	0.339	0.302
1cw	yes	0.224	0.251	0.404	0.533	0.224	0.251	0.404	0.224	0.251	0.224
2	yes	0.206	0.226	0.260	0.336	0.206	0.226	0.260	0.206	0.226	0.206
3	yes	0.206	0.226	0.259	0.336	0.206	0.226	0.259	0.206	0.226	0.206

Table 15

SEs ($\times 10$) of parameter estimates when n=1000, times between visits are divided by four and visits are regular and there is random censoring. 'Mtd' is method ('1cw' is Method 1 with censoring weighting) and 'Con' is whether constraint $\psi_{k(k+m)} = \psi_{k'(k'+m)}$ is imposed.

Mtd	Con	$\psi_{0(0)}$	$\psi_{0(1)}$	$\psi_{0(2)}$	$\psi_{0(3)}$	$\psi_{1(1)}$	$\psi_{1(2)}$	$\psi_{1(3)}$	$\psi_{2(2)}$	$\psi_{2(3)}$	$\psi_{3(3)}$
True		0.400	0.100	0.040	0.020	0.400	0.100	0.040	0.400	0.100	0.400
1	no	0.339	0.097	0.004	0.020	0.514	0.056	0.032	0.396	0.048	0.400
1cw	no	0.338	0.101	0.004	0.020	0.515	0.054	0.033	0.391	0.063	0.397
2	no	0.391	0.095	0.031	0.006	0.396	0.092	0.030	0.416	0.112	0.405
1	yes	0.402	0.075	0.019	0.020	0.402	0.075	0.019	0.402	0.075	0.402
1cw	yes	0.400	0.081	0.018	0.020	0.400	0.081	0.018	0.400	0.081	0.400
2	yes	0.399	0.099	0.030	0.007	0.399	0.099	0.030	0.399	0.099	0.399

Table 16

Means ($\times 10$) of parameter estimates when n=1000, times between visits are divided by four and visits are irregular and there is random censoring. 'Mtd' is method ('1cw' is Method 1 with censoring weighting) and 'Con' is whether constraint $\psi_{k(k+m)} = \psi_{k'(k'+m)}$ is imposed.

Mtd	Con	$\psi_{0(0)}$	$\psi_{0(1)}$	$\psi_{0(2)}$	$\psi_{0(3)}$	$\psi_{1(1)}$	$\psi_{1(2)}$	$\psi_{1(3)}$	$\psi_{2(2)}$	$\psi_{2(3)}$	$\psi_{3(3)}$
1	no	1.589	1.923	2.026	0.753	1.967	2.670	2.217	2.197	2.695	2.458
1cw	no	1.559	1.894	2.031	0.755	1.920	2.629	2.220	2.162	2.642	2.413
2	no	0.342	0.347	0.324	0.356	0.420	0.441	0.461	0.453	0.429	0.444
1	yes	1.021	1.312	1.512	0.729	1.021	1.312	1.512	1.021	1.312	1.021
1cw	yes	0.999	1.298	1.515	0.733	0.999	1.298	1.515	0.999	1.298	0.999
2	yes	0.201	0.233	0.266	0.354	0.201	0.233	0.266	0.201	0.233	0.201

Table 17

SEs ($\times 10$) of parameter estimates when n=1000, times between visits are divided by four and visits are irregular and there is random censoring. 'Mtd' is method ('1cw' is Method 1 with censoring weighting) and 'Con' is whether constraint $\psi_{k(k+m)} = \psi_{k'(k'+m)}$ is imposed.

$\mathcal{A}_0$	$\mathcal{A}_1$	Mtd	$\psi_{0(0)}$		$\psi_{0(1)}$		$\psi_{1(1)}$	
	True		0.300		0.100		0.300	
correct	correct	1	0.293	(0.000)	0.101	(0.002)	0.298	(0.001)
		2	0.295	(0.000)	0.101	(0.001)	0.301	(0.001)
		3	0.295	(0.000)	0.101	(0.001)	0.300	(0.001)
correct	misspec	1	0.292	(0.000)	0.104	(0.001)	0.262	(0.001)
		2	0.294	(0.000)	0.100	(0.001)	0.301	(0.001)
		3	0.294	(0.000)	0.100	(0.001)	0.302	(0.001)
misspec	correct	1	0.262	(0.000)	0.087	(0.001)	0.298	(0.001)
		2	0.298	(0.000)	0.104	(0.001)	0.302	(0.001)
		3	0.300	(0.000)	0.104	(0.001)	0.301	(0.001)
misspec	misspec	1	0.262	(0.000)	0.088	(0.001)	0.262	(0.001)
		2	0.298	(0.000)	0.102	(0.001)	0.300	(0.001)
		3	0.300	(0.000)	0.101	(0.001)	0.301	(0.001)

Table 18

Results from simulation study to investigate double robustness of Methods 2 and 3. Mean estimates ($\times -1$) over 5000 simulated datasets are shown, along with Monte Carlo standard errors of these means (in brackets). 'Mtd' means method. Numbers in red indicate estimators expected to be inconsistent.
