# Supplementary material for: Adjusting for time‐varying confounders in survival analysis using structural nested cumulative survival time models
Source: Biometrics. 2019 Nov 7;76(2):472–83. doi: 10.1111/biom.13158 (PMC7317577; doi:10.1111/biom.13158)
Supplement: Supplementary file 3 — Supplementary Information [file BIOM-76-472-s003.pdf]

Supporting Information

Web Appendices, Web Tables and R function referenced in Sections 4–7 and 9 are available with this paper at the Biometrics website on Wiley Online Library.
